# Investigation of Distinctive Morpho-Physio and Biochemical Alterations in Desi Chickpea at Seedling Stage Under Irrigation, Heat, and Combined Stress

**DOI:** 10.3389/fpls.2021.692745

**Published:** 2021-09-27

**Authors:** Saima Jameel, Amjad Hameed, Tariq Mahmud Shah

**Affiliations:** Nuclear Institute for Agriculture and Biology (NIAB), Faisalabad, Pakistan

**Keywords:** physiological markers, antioxidants, morphology, seedling, *Cicer arietinum*, yield, climate-smart

## Abstract

Global climatic instabilities have become the main reason for drastic yield losses in chickpea. This shift in climate could be a great threat in the future for food security in developing countries. Chickpea production is badly hampered by heat stress coupled with drought stress, and these factors can reduce yields by 40–45%. To mitigate yield losses due these abiotic factors, irrigation supplementation could be the best strategy. The present study aimed to (i) investigate the tolerance response of 9 desi chickpea genotypes against heat stress (H), irrigation (I), and a combination of both (I+H) through morphophysiological and biochemical indices at early growth stage, and (ii) assess yield performance across multiple locations of the country. Results revealed that under irrigation treatment, all genotypes perform well, but the genotypes D-09027 and D-09013 showed best performance because, as compared to control, they retained root length, seedling fresh weight, root fresh weight, root dry weight, esterase activity, Malondialdehyde (MDA) content, total chlorophyll, and total carotenoids. Shoot length and total phenolic contents (TPC) increased in both genotypes. Superoxide dismutase (SOD) and peroxidase (POD) increased in D-09027 and retained in D-09013. Catalase activity increased in D-09013 and retained in D-09027. Protease activity, total water potential and osmotic potential decreased in both genotypes and depicted high yield potential with 27 and 30% increase in yield over Bhakhar-2011 (check), respectively. In case of heat stress, maximum tolerance was found in genotypes CH104/06 and D-09013 with no change in shoot and root length, seedling dry weight, shoot fresh and dry weight, root dry weight, relative water content, turgor water potential, catalase (CAT) activity, esterase activity, increased root fresh weight, peroxidase activity (POD), ascorbate peroxidase activity (APX), and lycopene with low accumulation of protease and Malondialdehyde content (MDA). Both genotypes depicted high yield potential with 30 and 43% increase in yield over check across multiple locations of the country. Under the combined treatment, most genotypes showed good performance, while CH104/06 was selected as best performer genotype because significant of its increased root fresh weight, lycopene content, chlorophyll b, total carotenoids, total chlorophyll, retained shoot length, root length, seedling fresh and dry weight, total water potential, osmotic potential, relative water content, peroxidase activity (POD), catalase, esterase, and its ascorbate peroxidase (APX) activity and total soluble proteins (TSP) showed highest yield potential with 43% increase over check. Identified best performing and tolerant genotypes can further be employed for breeding climate-smart chickpea genotypes for sustainable production under changing climate.

## Introduction

Legumes are very important in human diet; they are not only complement nutrients (minerals, carbohydrates) to the cereal diet but also improves the texture and taste of staple dish (Zia-Ul-Haq et al., 2007; Varol et al., 2020). Legumes are a member of the family Fabaceae, which has up to 750 genera and 20,000 species (Graham and Vance, 2003). Chickpea (*Cicer arietinum* L.) is a very important member of legumes. Also known as Bengal Gram or Garbanzo, it is ranked third in number after dry beans and peas worldwide (Jaiswal et al., 2012; Sivasakthi et al., 2017; Yegrem, 2021). They are old world pulses and have a nut like flavor with numerous applications in food. They were considered to originate from levant and ancient Egypt (Wallace et al., 2016), but some studies suggest that they originated from Turkey (Faridy et al., 2020). Chickpea is divided into two distinctive groups, i.e., desi type (microsperma) and Kabuli type (macrosperma), which differs in terms of seed morphological traits (Moreno and Cubero, 1978). Both desi and kabuli are nutrition-rich grain legumes and are two of the inexpensive sources of protein as compared to animal protein, and are therefore crucial for nutritional security in developing countries, especially in Pakistan (Iquebal et al., 2017). Chickpea also serves as a soil fertility enhancer via biological nitrogen fixation process and fits in various crop rotations to improve soil fertility (Thudi et al., 2017; Kaur et al., 2020). Chickpea is grown in over 59 countries spanning India, Pakistan, North Africa, Middle East, America, Australia, and southern Europe, etc. Asia alone produces 87% of the overall world production (Lev-Yadun et al., 2000). Globally, chickpea occupies 14.56 Mha area and, recently, 14.78 MT production was recorded (FAO STAT 2019) (Kumar et al., 2021). The top chickpea producing countries are India, Pakistan, Turkey, Australia, and Myanmar with India as the largest chickpea producing country, producing 70% of the total production of the world (Wallace et al., 2016; Kushwah et al., 2021). Desi type is more important, mostly grown in Asian and African countries, it covers about 80–85% of world chickpea production (Pande et al., 2005). The major share in the world's chickpea production is from Asian countries in which India and Pakistan are major suppliers (Sharma and Sharma, 2020). Chickpea is a rabi crop requiring an optimal temperature range of 10–35°C day and night, respectively, for normal plant growth, grown particularly in the arid and semi-arid regions by farmers, where water requirements are achieved either with seasonal rainfall or stored soil moisture (Shah et al., 2020; Sahu et al., 2021). Chickpeas are rich source of high quality proteins, comprised of albumins and globulins in a large quantity (Saharan and Khetarpaul, 1994; Nasir et al., 2017). Desi chickpea cultivars serve as potential sources of nutritional components, i.e., amino acid, essential fatty acids, trace elements, and minerals (P, Ca, K, Zn, Cu, Mg, etc.) (Duke, 2012).

Pakistan can be divided into three ecological zones according to chickpea production. Northern areas are with high rainfall while the central region has fertile soil but has a semi-arid climate. Chickpeas are also grown under rain-fed conditions, i.e., in southern Punjab, where annual rainfall pattern is quite flimsy and agriculture is totally dependent upon rain (Khan, 1990). Province wise chickpea is grown 82% in Punjab, 9% in Khyber Pakhtunkhwa, 8% in Sindh, and 1% in Baluchistan. In Punjab, 90% of chickpea is grown under rain fed conditions. The thal region alone shares about 80% of the country's chickpea production (Shah et al., 2007). Different abiotic stresses are the major threats for crop production and food security globally (Mach et al., 2019).

Recently, drastic fluctuations in climatic events and weather extremes have been observed in different parts of the world. In different parts of Asia, Europe, and Australia, the frequency of heat waves has increased (Venkatramanan and Shah, 2019). Moreover, climatic changes are imposing high pressure on the hydrological cycle (Kang et al., 2009). From the last few years, unpredicted changes in climatic conditions, resulting in high global temperature (heat stress) and unusual rainfall patterns (floods and drought) are becoming major challenges for chickpea production (Shafiq et al., 2021). Such weather extremes badly affect plant growth and resultantly lowers crop yield, particularly in chickpea grain yield where losses were observed up to 19% due these factors (Kadiyala et al., 2016). It was reported that it is detrimental for lowering yield potential at reproductive stage of chickpeas if day temperature exceeds from 30°C (Kaushal et al., 2013). It is studied that vegetative and reproductive growth stages of wheat, rice, cotton and chickpeas are affected by heat stress (Kushwah et al., 2021). Low moisture and high temperature stresses are the most important yield limiting constraints among climate events (Ksiezak and Bojarszczuk, 2020). The effect of irrigation treatment or heat stress on any plant depends upon certain factors, i.e., intensity and duration of irrigation supplementation and heat stress, on specific genotype, and its developmental stage (Simova-Stoilova et al., 2008). It is estimated that 50% of yield losses are caused by drought and about 15–20% yield losses for heat stresses in chickpeas (Varshney et al., 2014). Combined stress of drought and heat at flowering and pod filling stage of chickpea can minimize grain yield by 40–45% (Rani et al., 2020). In chickpeas, yield losses of about 10–15% have been reported due to every 1°C increase above the optimum temperature required for normal plant growth (Devasirvatham and Tan, 2018). It has been reported that the main cause for low yield is loss of pollen viability under heat stress globally as chickpea is a heat sensitive crop (Bhandari et al., 2020; Pattison et al., 2021). Globally, most of the chickpea crop is produced on residual soil moisture but supplemental irrigation can boost the production (Ali, 2017). In some parts of the world, especially in west Asia, strategies to overcome the yield losses due to terminal drought and heat stress in chickpea includes cultivating it with irrigation supplementation to amplify massive chickpea production (Khamssi et al., 2010; Erman et al., 2011; Singh et al., 2016). Irrigation supplementation at early pod filling and during the pre-flowering stage caused an increased yield in chickpeas (Saxena, 1980; Varol et al., 2020). Irrigation application not only increased yield but also minimized the risk of crop failure in dry years with reduced or no rainfall in chickpea growing regions (Silva et al., 2014).

Under different abiotic stresses, plants face various morph physiological and biochemical alterations at cell level for plant adaptation (Ghiabi et al., 2013; He et al., 2018; Saikia et al., 2021). During environmental stresses, plants endure oxidative stress by overproduction of reactive oxygen species (ROS) which are highly toxic and reactive molecules (oxides, superoxides, etc.) which cause severe injuries to lipids, carbohydrates, proteins, and DNA that ultimately causes cell death (Gechev and Petrov, 2020; Kumari et al., 2021). Plants counteract ROS with oxidative stress through endogenous defense mechanism consisting enzymatic [superoxide dismutase (SOD); peroxidase (POD); catalase (CAT); ascorbate peroxidase (APX) etc.] and non-enzymatic antioxidants (ascorbic acid, carotenoids, phenolic contents, glutathione etc.) (Kaur et al., 2019; Zafar et al., 2019; Hasanuzzaman et al., 2020). In chickpea, heat tolerance mechanisms are associated with elevated production of antioxidants and osmolytes, which helps to sustain metabolism, protect macromolecules, retain membrane integrity leading to acclimation under heat stress (Yadav et al., 2012; Parankusam et al., 2017; Rani et al., 2020). In several economically important crops (wheat, rice, cotton), the effect of heat stress on vegetative and reproductive growth period was studied through morphological and physiological approaches. However limited research was done in chickpeas (Kumari et al., 2018; Prajapat et al., 2018; Kushwah et al., 2021). Climate-smart agriculture appears to be a credible approach to minimize the harsh effect of climate change on the adaptation of plants (Kumari et al., 2020).

To date, limited research is available in desi chickpea at the seedling stage for above mentioned abiotic factors. Therefore, the present study was planned with the following main objectives: (i) to assess the effect of irrigation, heat stress, and the combination of both heat stress and irrigation on morphology, physiology, and biochemistry of desi chickpea at the seedling stage, and (ii) to assess yield potential across multiple locations of the country. Moreover, it would help in the rapid screening of best performing genotypes for irrigation (I), heat (H) and combined stress (I+H) for subsequent utilization in chickpea breeding programs. Identified best performing and tolerant genotypes can further be employed for breeding climate-smart chickpea genotypes for sustainable production under a changing climate.

## Materials and Methods

### Genetic Material

Healthy and disease-free seeds of nine desi chickpea genotypes were obtained from different research institutes of Pakistan to conduct the present study (Table 1).

**Table 1:** List of genotypes along with their source used in the study.

**Sr. No**.	**Genotypes**	**Source**	**Sr. No**	**Genotypes**	**Source**
1	D-09013	AARI Faisalabad	6	BRC−390	RARI, Bahawalpur
2	D-09027	AARI Faisalabad	7	CMC 211S	NARC, Islamabad
3	CH 24/07	NIAB Faisalabad	8	NIFA−2	NIFA, Peshawar
4	CH104/06	NIAB Faisalabad	9	Bhakhar-2011	AZRI, Bhakkar (Check)
5	TGX 225	AZRI, Bhakkar			

### Experiment Plan

The experiment was conducted in a completely randomized design (CRD) with four replications to investigate the effect of treatments on morphology, physiology, and biochemistry of desi chickpea at seedling stage during the year 2015–2016. The experiment was performed under the net house conditions of the Nuclear Institute for Agriculture and Biology (NIAB), Faisalabad, where the average day and night temperature was 10 and 32°C, respectively. Round pots with specific dimensions (top width, 27 cm; bottom width, 20.5 cm) were used for the present study. There were four sets of 36 pots in each: one set for control, the second set for irrigation, the third set for heat stress, and the fourth set for combined stress application. For this purpose, round pots (top width, 27 cm; bottom width, 20.5 cm) were filled with 3 kg of autoclaved sieved pure sand. The water holding capacity of the sand was measured which was 25%. To attain field capacity, 750 ml of distilled water was added to each pot. Twelve seeds of each genotype were sown ~5–8 cm depth in sand filled pot.

### Irrigation Treatment

**Figure 1:**
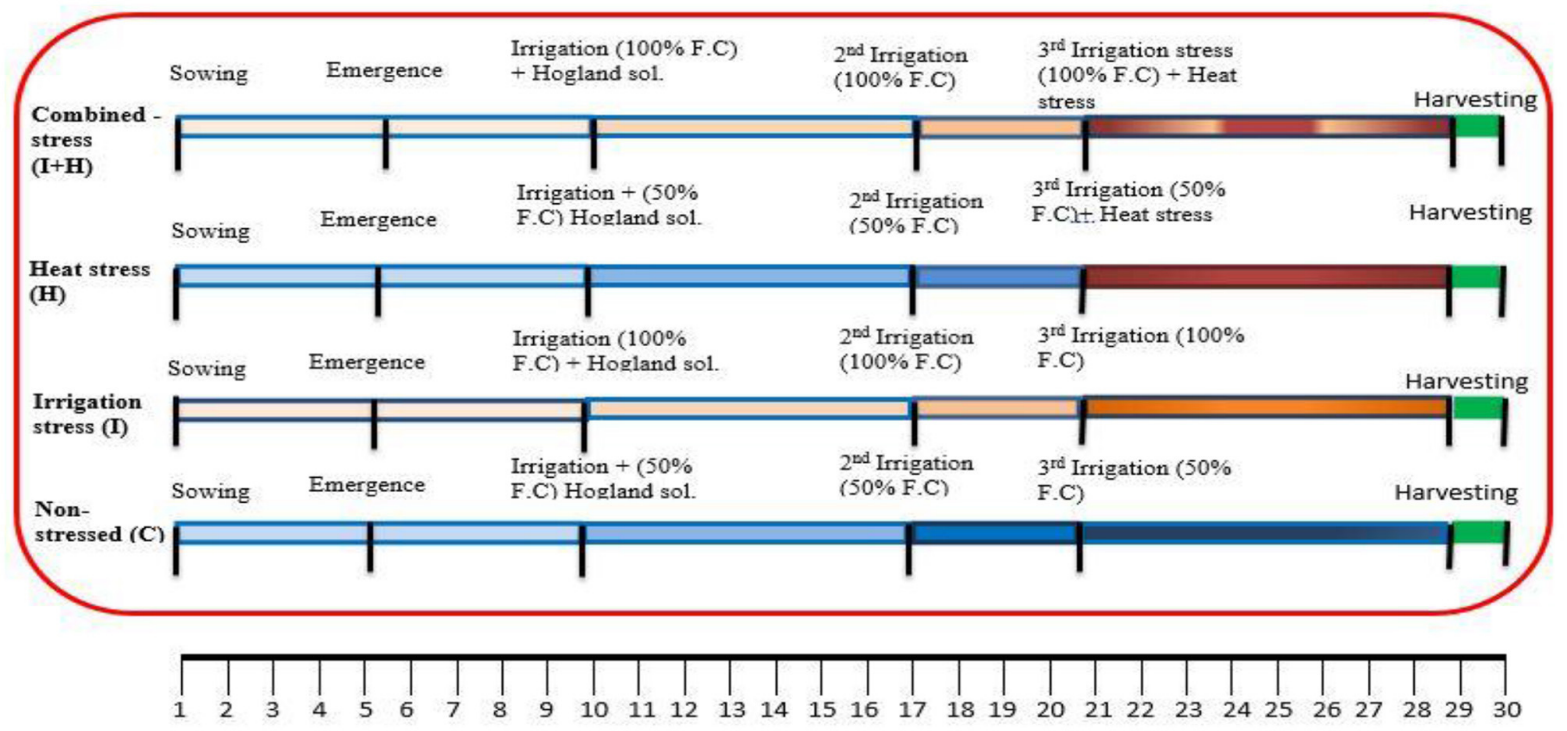
Scheme diagram of protocol followed for screening against irrigation (I), heat (H), and combined (I+H) stress on Chickpea seedlings, irrigation stress at 100% field capacity, heat stress 38/17°C, combined stress (38/17°C+100% F.C), 1/8th strength Hoagland Solution.

After 5–6 days of the sowing date, seeds were germinated. Hoagland solution of 1/8^th^ strength was applied on the 10th day of sowing to each set of an experiment to attain the plant's essential nutritional requirements. The controlled and heat-stressed pots were irrigated at half of their field capacity on the 5th day after seed germination, while irrigated and combined stressed pots were irrigated at full of their field capacity on the same day. Second and third irrigations were applied according to the above-mentioned scheme on the 17th and 21st days of sowing (Figure 1).

### Exposure of Heat Stress at Seedling Stage Under Glasshouse Conditions

After 2 weeks of germination, two sets of pots, one for heat stress and the other for combined stress were shifted in the glasshouse of NIAB for heat stress exposure for 10 days, where the average day and night temperature was sustained at 38 and 17°C, respectively, by using electric bulbs with a light intensity at 2,500 l x for 14 h. The experiment was harvested on the 30th day of sowing (Figure 1).

### Physiological Parameters

#### Leaf Water Potential

Leaf water potential was measured with the help of scholander pressure chamber (Arimad, UK). The same leaf sample was used for osmotic potential measurement by vapor pressure osmometer (wescor 5500). Leaf turgor pressure was calculated as the difference between leaf water potential and leaf osmotic potential values (Grieve and Grattan, 1983).

#### Relative Water Content

For measurement of RWCs, the leaf samples were detached, and their fresh, dry, and saturated weights were calculated by using a sensitive weighing balance. The RWC was calculated with the following formula (Weatherley, 1950; Kumar et al., 2013).


$$\begin{array}{l} \text{RWC }=\;\frac{\left(\text{leaf fresh weight }-\text{ leaf dry weight}\right)}{\left(\text{Leaf saturated weight }-\text{ leaf dry weight}\right)}\;\times \;100 \end{array}$$

#### Chlorophyll Florescence

Chlorophyll fluorescence was detected by a portable fluorescence spectrometer “plant efficiency analyzer” (PEA, Handsatech instruments Ltd.) (Strasserf and Srivastava, 1995). After a dark adaptation period of 30 min using specific leaf clips, the minimal fluorescence level (Fo), with all photosystem II (PSII) reaction centers open, was measured by a weak red light which was sufficiently low (<0.1 μmol m^−2^s^−1^) to prevent inducing any significant variable fluorescence. All measurements of Fo were performed with the measuring beam set to a frequency of 6,000 Hz, whereas all measurements of F_m_ were performed with the measuring beam automatically switching to 20 kHz during the saturating flash. All the parameters were recorded according to a previous study (Litchtenthaller and Wellburn, 1985). From these parameters, the following characteristics were also recorded: minimal chlorophyll fluorescence (Fo) of a dark-adapted leaf, maximal chlorophyll fluorescence (F_m_) of a dark-adapted leaf, variable chlorophyll fluorescence (F_v_) of the dark-adapted leaf, time of achieving maximum fluorescence yield (T_m_), the ratio of variable chlorophyll fluorescence, and minimal chlorophyll fluorescence *Fv*/*Fm*.

### Biochemical Analysis

All biochemical analyses including enzymatic, non-enzymatic, hydrolytic antioxidants, and certain other biochemical assays were performed at Marker Assisted Breeding Lab-1 Plant Breeding and Genetics Division, Nuclear Institute for Agriculture, and Biology (NIAB), Faisalabad, Pakistan.

#### Extraction of Sample

Fresh leaves (0.2 g) of all chickpea genotypes were ground in 2 ml of 50 mM cold phosphate buffer (pH 7.8) and centrifuged at 15,000 × g for 20 min at 4°C. The supernatant was used for the determination of enzymatic, non-enzymatic, hydrolytic antioxidants, and other biochemical parameters. The analysis was performed in four repeats for all treatments including control.

### Enzymatic Antioxidants

#### Superoxide Dismutase Activity

For Superoxide dismutase activity (SOD) determination, chickpea leaves were homogenized in a medium composed of 50 mM potassium phosphate buffer (pH 7.8), 0.1 mM EDTA, and 1 mM dithiothreitol (DTT) as described by. The activity of SOD was assayed by measuring its ability to inhibit the photochemical reduction of nitro blue tetrazolium (NBT) following a previous method (Giannopolitis and Ries, 1977). One unit of SOD activity was defined as the amount of enzyme which caused 50% inhibition of photochemical reduction of NBT.

#### Peroxidase Activity

In order to analyze peroxidase activity (POD), chickpea leaves were ground in a medium composed of 50 mM potassium phosphate buffer (pH 7.0),0.1 mM EDTA and 1 mM DTT. The activity of POD was measured using the method of Chance and Maehly (1955) with some modification. For measurement of POD activity, assay solution (3 mL) contained 50 mM phosphate buffer (pH 7.0), 20 mM guaiacol, 40 mM H_2_O_2_ and.1 ml enzyme extract. The reaction was initiated by adding the enzyme extract. An increase in absorbance of the reaction solution at 470 nm was recorded every 20 s. one-unit POD activity was defined as an absorbance change of.01 units/min.

#### Catalase Activity

For the estimation of CAT, leaves were homogenized in a medium composed of 50 mM potassium phosphate buffer, pH 7.0, and 1 mM DTT. CAT was estimated by the following method described by Chance and Maehly (1955). For measurement of CAT activity, 5.9 mM H_2_O_2_ and.1 ml enzyme extract. A decrease in absorbance of the reaction solution at 240 nm was recorded every 20 s. An absorbance change of 0.01 units min^−1^ was defined as one-unit CAT activity. Enzyme activities were expressed on a fresh weight basis.

#### Ascorbate Peroxidase Activity

To determine the ascorbate peroxidase activity, chickpea leaves were emulsified in a medium composed of 50mM potassium phosphate buffer (pH 7.0). APX activity was measured using the method of Dixit et al. (2001). Assay buffer was prepared by mixing 200 mM potassium phosphate buffer (pH 7.0), 10 mM ascorbic acid, and.5 M EDTA. For measurement of APX, the activity assay solution contained an assay buffer made up of 10 mM ascorbic acid 0.5 M EDTA, and 200 mM potassium phosphate buffer, H_2_O_2_ (1 ml), and supernatant 50 μl. The oxidation rate of ascorbic acid was estimated by following the decrease in absorbance at 290 nm after every 30 s (Chen and Asada, 1989).

### Non-enzymatic Antioxidants

#### Total Phenolic Contents

Micro colorimetric technique (Ainsworth and Gillespie, 2007) was used for the total phenolic assay in chickpea leaves, which utilizes Folin-Ciocalteu (F-C) reagent. A standard curve was prepared using different concentrations of gallic acid and a linear regression equation was calculated. Phenolic content (gallic acid equivalents) of samples was determined by using the linear regression equation.

### Hydrolytic Enzymes

#### Esterase Activity

Method described by van Van Asperen (1962) was used, with some minor modifications for esterase activity estimation in chickpea leaves. A leaf sample of 500 mg homogenized in 5 ml phosphate buffer (100 mM, pH7.0), containing 1 mM of each EDTA, PMSF, PTU, and 20% glycerol by using mini bead beater and then centrifuged at 10,000 × g for 20 min at 4°C. The supernatant was used for enzyme estimation. Different standards (0.1 to.9 μM interval) of an esterase from a working stock solution were prepared in 1,000 μl distilled water. Then, 5 ml of phosphate buffer (40 mM, pH 6.8) were added to all of the standard solutions including blank before finally adding 1 ml of staining solution to each and placed them for 20 min in the dark at 30°C for incubating with gentle shaking. Absorbance at 590 nm was measured. Standard curves for a-naphthyl acetate esterase were prepared by using standards.

#### Protease Activity

To analyze the protease activity, the leaf sample was homogenized in a medium composed of 50 mM potassium phosphate buffer (pH 7.8). Protease activity was determined by the casein digestion assay described by Drapeau (1974). By this method, one unit is that amount of enzyme, which releases acid-soluble fragments equivalent to.001 A280 per minute at 37°C and pH 7.8. Enzyme activity was expressed on a fresh weight basis.

### Other Biochemical Parameters

#### Total Oxidant Status

Total oxidant status was determined by using a formulated method (Erel, 2005) which is based on the oxidation of ferrous ion to ferric ion by oxidants present in the sample in an acidic medium and the measurement of ferric ion by xylenol orange (Harma et al., 2005). The assay mixture contained reagent R_1_, reagent R_2_, and sample extract. After 5 min, the absorption was measured at 560 nm by using a spectrophotometer. A standard curve was prepared using hydrogen peroxide. The results were expressed in μM H_2_O_2_ equivalent per liter.

#### Malondialdehyde Content

The level of lipid peroxidation in the leaf tissue was measured in terms of (MDA, a product of lipid peroxidation) content determined by the thiobarbituric acid (TBA) reaction using the method of Zhang and Kirkham (1994). A 25 g leaf sample was homogenized in 5 ml.1% TCA. Homogenate was centrifuged at 10,000 × g for 5 min. In 1 ml aliquot of the supernatant, 4 ml of 20% TCA containing.5% TBA were added. The mixture was heated at 95°C for 30 min and then quickly cooled in an ice-bath. After centrifuging at 10,000 × g for 10 min, the absorbance of the supernatant at 532 nm was read and the value for the non-specific absorption at 600 nm was subtracted. The MDA content was calculated by using an extinction coefficient of 155 mM ^−1^ cm^−1^.

#### Total Soluble Proteins

For TSP estimation in chickpea leaves, 5 μl of supernatant and 95 μl 150 mM NaCl were mixed with 1.0 ml of dye reagent (100 mg). Coomassie Brilliant Blue G-250 dye was dissolved in 50 ml of 95% ethanol and 100 ml 58% (w/v) phosphoric acid and diluted to 1 L and the mixture was left for 5 min to form a protein-dye complex. Then the absorbance was measured at 595 nm (Bradford, 1976).

#### Pigment Analysis

The amount of Pigments (total chlorophyll, chlorophyll a, chlorophyll b, lycopene and carotenoids) was determined by using method given (Lichtenthaler and Wellburn, 1983). Samples (0.2 g) were ground in acetone (80%) and centrifuged at 10,000 × g for 5 min. Absorbance measured at 663, 645, 505, 453, and 470 nm wavelength by using a spectrophotometer (SPH-003).

### Field Performance of Genotypes Under Multiple Environments

**Figure 2:**
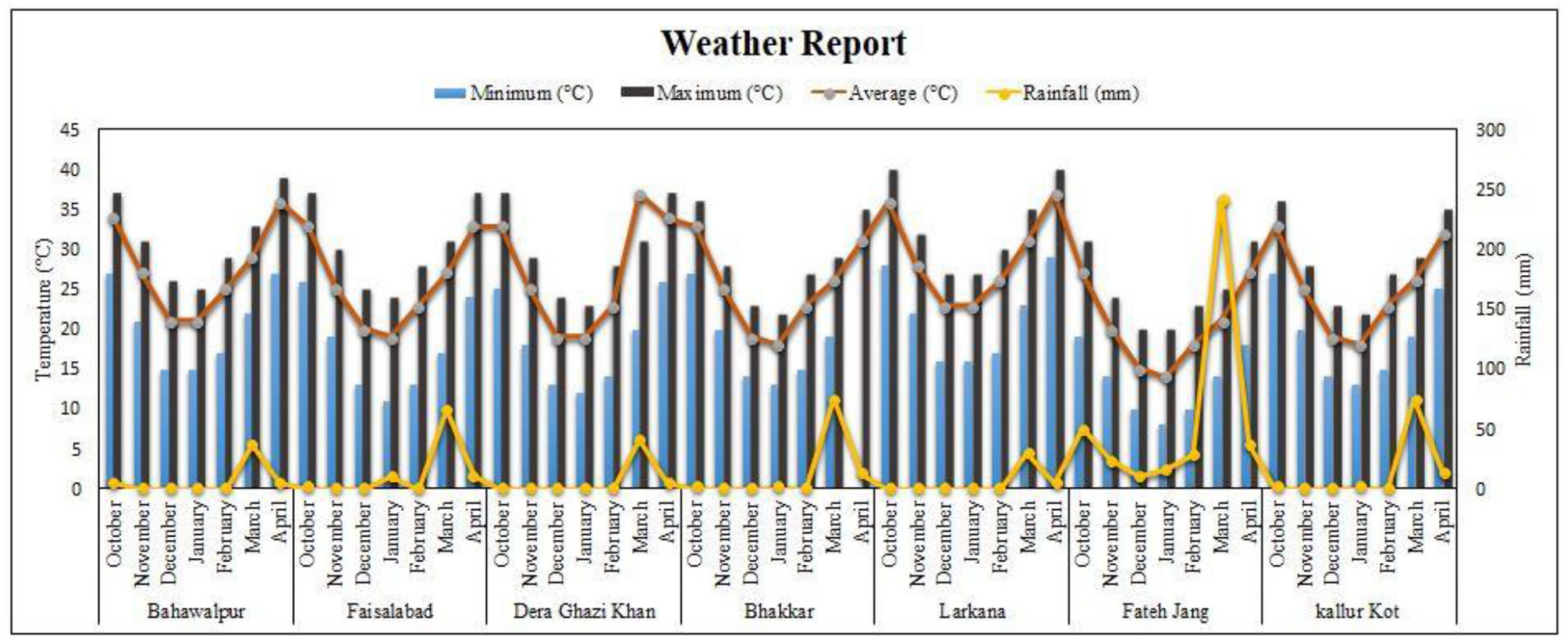
Description of weather Conditions for all locations (year 2015–2016).

To check the yield performance of these lines national uniform yield trials were performed across the country. Experiments were conducted in randomized complete block design with three replications in each environment. Eight locations were targeted for this purpose, namely, 1 = NIAB, Faisalabad, 2 = PRI, AARI, Faisalabad, 3 = GBRS, Kalurkot, 4 = BARS, Fateh Jang, 5 = AZRI, Bhakkar, 6 = QAARI, Larkana, 7 = RARI, Bhawalpur, and 8 = AZRI, D.I.Khan. Bhakhar-2011 and Pb-2008 were used as checks for the evaluation of yield performance. Weather data from October to April for targeted places were collected from https://www.worldweatheronline.com (Figure 2). Average yield data was collected from all the targeted environments.

### Statistical Analysis

The screening experiments were conducted in four repeats using a completely randomized design (CRD). The significance was ascertained by ANOVA and Tukey (HSD) test at *p* < 0.05 by using XLSTAT 2014.5.03 software. To check the response of the genotype under different treatments, bar graphs were constructed based on mean ± S.E. In graphs, bars with different alphabets were significantly different from each other. Principle component analysis (PCA) for most reliable genotypic selection under different conditions along with control was performed, and for the first two principal components (PC-I and PC-II) biplots were constructed by using the same software. Correlation (Pearson test) and cluster analysis was also performed by algometric hierarchical clustering for all genotypes under all treatments by using the same software.

## Results

### Morphological Parameters

#### Shoot and Root Length

In comparison with non-stress conditions, significant genotypic differences were observed for shoot length under different stresses (Supplementary Figure 1). Under irrigation treatment, a significant increase in shoot length was observed for all genotypes with exception of TGX 225 which remained unchanged. Overall, the highest increase in seedling length was found in CH104/06 followed by D-09027. In the case of heat stress, the shoot length of four genotypes (D-09027, CH 24/07, TGX 225, and CMC 211S) decreased significantly, while others were not affected significantly. When shoot length was measured under the combination of heat with irrigation treatment, it was noticed that shoot length increased significantly in D-09013, BRC-390, CMC 211S, and Bhakhar-2011, while others were showing non-significant changes. Irrigation treatment significantly increased root length only in genotype CH 24/07, while it was retained in others as compared to non-stress condition. Root length was not affected by heat stress and combined stress treatment with the exception of Bhakhar-2011, in which root length was significantly increased compared to that under control (Supplementary Figure 1).

#### Seedling Fresh and Dry Weights

Under irrigation and heat stress, seedling fresh weight was not affected significantly in all genotypes, except D-09013 in which a significant increase in seedling fresh weight was observed as compared to control. Under combined stress, a significant increase in seedling fresh weight was observed in BRC-390 followed by Bhakhar-2011 (Supplementary Figure 1) while it remained unchanged in others. In the case of irrigation, a significant increase in seedling dry weight was observed in BRC-390, TGX 225, and Bhakhar-2011, while it remained unchanged in others. Under heat and combined stress, a significant increase in seedling dry weight was observed only in BRC-390, while the remaining genotypes showed a non-significant change (Supplementary Figure 1).

#### Shoot Fresh and Dry Weights

In comparison with non-stress conditions, under irrigation, a significant increase in shoot fresh weight was only observed in D-09013 and CH 24/07, while the shoot fresh weight of all other genotypes was not affected by irrigation treatment. In case of heat stress, shoot fresh weight was retained in all genotypes as compared to control conditions, except TGX 225 in which shoot fresh weight was significantly decreased. Combined stress significantly increased shoot fresh weight in BRC-390 and CH 24/07, while all others were showing non-significant behavior toward this stress (Supplementary Figure 2). Shoot dry weight was significantly increased in D-09013 and CH 24/07 while in leftovers, it remained unchanged under irrigation. Shoot dry weight was not affected in all genotypes in heat and combined stress conditions, while BRC-390 showed a significant increase in shoot dry weight (Supplementary Figure 2).

#### Root Fresh and Dry Weight

Under irrigation, root fresh weight was significantly increased only in BRC-390 and Bhakhar-2011 while no change was revealed by all other genotypes. Under heat stress, root fresh weight of most of genotypes was retained, with the exception of D-09027 in which significant decrease was noticed. D-09013 showed increased root fresh weight in comparison to that of non-stressed conditions. Combined stress significantly increased root fresh weight of BRC-390 and CH104/06, while the non-significant change was recorded for all other genotypes (Supplementary Figure 2). Significant increase in root dry weight was observed in BRC-390 followed by Bhakhar-2011 under irrigation, while all other genotypes depicted a non-significant change. Root dry weight was not affected in all genotypes under heat and combined stress, except D-09013 which showed a significant decrease under combined conditions compared to that of control (Supplementary Figure 2).

### Physiological Parameters

#### Total Water Potential

In comparison with non-stress conditions, significant differences were observed for leaf total water potential under different stresses (Supplementary Figure 3). Under irrigation treatment, significant decreasing behavior was observed in total water potential for all genotypes. In the case of heat stress, total water potential was generally increased in genotypes D-09013, D-09027, and NIFA-2 as compared to control conditions, while a significant decrease was observed in BRC-390 and Bhakhar-2011 but retained in leftovers. When total water potential was recorded under combined stress of heat and irrigation, it was found that a non-significant change in total water potential was found in all genotypes except BRC-390 followed by Bhakhar-2011.

#### Osmotic Water Potential

Osmotic water potential was significantly decreased in most genotypes. Despite this, osmotic water potential increased significantly in CH104/06 and CMC 211S and was retained under irrigation treatment in NIFA-2. Heat stress caused a significant decrease in osmotic water potential in D-09013 BRC-390 and Bhakhar-2011, while an increase in osmotic potential was induced in all others. Combined stress caused a significant decrease in osmotic potential in all genotypes (Supplementary Figure 3).

#### Turgor Potential and Relative Water Content

In irrigation, turgor potential was significantly increased in D-09013, CH24/07, CH104/06, and CMC 211S, while a significant decrease was shown by TGX 225 and BRC-390. In leftovers, it was found non-significant. Turgor potential was decreased significantly in TGX 225, while a significant increase was observed for D-09027, NIFA-2, CH24/07, CH104/06, and in Bhakhar-2011 but remained in heat stress in others. Under combined stress, a significant decrease in turgor potential was observed for all genotypes except in BRC-390 and CH24/07 in which no affect was found (Supplementary Figure 3). Overall, a significant increase in relative water content was observed for all genotypes under irrigation treatment, compared to that of non-stressed condition. In the case of heat stress and combined stress conditions, the relative water content of all genotypes was not affected (Supplementary Figure 3).

#### Chlorophyll Fluorescence

No significant differences were observed for fluorescence in the absence of light (Fo), maximum fluorescence (Fm), variable fluorescence (Fv), and Tm and *Fv*/*Fm* under all stresses in almost all genotypes compared to that of control (Supplementary Figures 4, 5).

### Biochemical Parameters

#### Enzymatic Antioxidants

##### Superoxide Dismutase

Irrigation treatment caused a significant increase in SOD activity in BRC-390, and Bhakhar-2011, however, it caused significant decreased SOD activity in CH 24/07 and TGX 225, while no effect was observed in leftovers as compared to non-stress condition. Under heat stress, SOD activity was decreased in D-09013, CH104/06, CH 24/07, TGX 225, and CMC 211S, while a significant increase was observed in Bhakhar-2011 and D-09027. No change was observed in the remaining samples. Under combined stress, significantly decreased activity was observed in all genotypes, except in CMC 211S and NIFA-2 which remained unchanged (Supplementary Figure 5).

##### Peroxidases Activity

Irrigation treatment significantly increased plant POD activity in D-09013, D-09027, CH104/06, and Bhakhar-2011. However, a significant decrease in POD activity was observed in CH24/07 and TGX 225 while it was retained in all other genotypes compared to that of control conditions. POD activity, in case of heat stress, was increased in CH104/07, D−09027, and Bhakhar-2011, while a significant decrease was found in CH 24/07 and TGX 225. No change was observed in all the others. Under combined stress, a significant increase in POD activity was observed in D-09027, CH 24/07, and Bhakhar-2011, while non-significant change was observed in all the others in comparison with the control (Supplementary Figure 5).

##### Catalase

Catalase activity was retained under irrigation in all the genotypes with exception of D-09013 and BRC-390 in which a significant increase was observed. Under exposure to heat stress, CAT activity was increased significantly in D-09013, Bhakhar-2011, BRC-390, and CH 24/07, while non-significant variations were observed in all other genotypes. Under the combined stress, a significantly increased CAT activity was noticed only in CH104/06, while it remained unchanged in all the other genotypes as compared to non-stress condition (Supplementary Figure 5).

##### Ascorbate Peroxidase

Ascorbate peroxidase activity was not affected significantly in most of the genotypes apart from D-09013 and CH104/06 in which a significant increase was observed in APX. D-09027 showed a significantly decreased activity under irrigation treatment. Under heat stress, compared with non-stress conditions, APX activity was increased in D-09013 and CH 24/07, while no affect was observed in all other genotypes. Under combined stress, a significant increase in APX activity was observed in D-09013 and Bhakhar-201, while all others showed non-significant behavior (Supplementary Figure 6).

#### Non-enzymatic Antioxidants

##### Total Phenolic Contents

Under irrigation, it was found that TPC decreased significantly in CH 24/07, BRC-390, NIFA-2, and in Bhakhar-2011, while increased in D-09013, D-09027, and CH104/06 while it remained unchanged in leftovers. In case of heat stress, it was detected that TPC was significantly decreased in all genotypes as compared to non-stress conditions. Under combined stress, TPC was decreased in D-09027, CH 24/07, CMC 211S, and NIFA-2, while combined stress showed no effect on TPC in other genotypes (Supplementary Figure 6).

### Hydrolytic Enzymes

#### Esterase Activity

Under irrigation treatment, esterase activity was significantly increased in CH104/06 and NIFA-2 while it decreased significantly in CH24/07 and Bhakhar-2011. It remained unchanged in all other genotypes. As compared to non-stress conditions, under exposure to heat stress, esterase activity was significantly increased only in BRC-390 and decreased in Bhakhar-2011. However, it remained unchanged in leftovers. Esterase activity was non-significant in all genotypes under Combined stress conditions with the exception of Bhakhar-2011, in which a significant decrease was observed (Supplementary Figure 6).

#### Protease Activity

As compared to non-stress conditions, protease activity was significantly decreased generally in all genotypes, except CH104/06 in which no change in protease activity was found, while least protease activity was observed in Bhakhar-2011 under irrigation. In heat stress, protease activity was significantly decreased in all genotypes, however, the least activity was found in Bhakhar-2011. Under combined stress, a significant increase in protease was observed only in CH104/06 while a significant decrease was found in all other genotypes as compared to non-stress conditions (Supplementary Figure 6).

### Other Biochemical Parameters

#### Total Oxidant Status

Total oxidant status was significantly increased in D-09013, D-09027, CH104/06, and TGX 225 under irrigation, while it decreased significantly in BRC-390 only, a non-significant increase was observed in all other genotypes. In case of heat stress, TOS of most of the genotypes was generally retained except CH104/06 and Bhakhar-2011 in which significant increase and decrease were found, respectively. Under combined stress, TOS value increased significantly in D-09013, CH104/06, and BRC-390 (3,433 ± 105 μM/g), while a significant decrease was observed in, Bhakhar-2011 and leftovers showed no change (Supplementary Figure 7).

#### Malondialdehyde Content

As compared to the control condition, under irrigation, MDA varied non-significantly in most genotypes except for BRC-390 in which a significant increase was observed. Significant decrease was observed in D-09013 and CH104/06. MDA content was generally not affected by combined stress application, except a significant increase in MDA contents was noticed in NIFA-2 (Supplementary Figure 7).

#### Total Soluble Proteins

Total soluble proteins under irrigation treatment were significantly increased in D-09013 and CMC211S. Significant decrease was observed in CH104/06, TGX 225, BRC-390 and Bhakhar-2011 while it remained unchanged in D-09027, CH 24/07 and NIFA-2. Heat stress only caused significantly increased TSP in D-9013. However, significant decreased TSP was observed in D-09027, CH104/06, TGX 225, BRC-390, and CMC 211S while it remained unchanged in others. Combination of both stresses induced significant decreases TSP in most of the genotypes except D-09013 which showed a marked increase in TSP, while 2 genotypes (TGX 225, Bhakhar-2011) retained TSP (Supplementary Figure 7).

### Pigment Analysis

#### Lycopene Contents

Irrigation treatment caused significant increased lycopene content in CH104/06 and CMC 211, while significantly decreased in D-09013 and D-09027, but retained in remaining genotypes compared to non-stressed conditions. In the case of heat stress, significant decrease in lycopene was observed in D-09013, D-09027, CH 24/07, and TGX 225, while it significantly increased in CMC 211, CH104/06, and Bhakhar-2011 but retained in reaming genotypes. Combined stress caused significant increase in CH104/06 and Bhakhar-2011 but significantly decreased in D-09013, CH 24/07, and BRC−390. It was retained in left over genotypes (Supplementary Figure 7).

#### Chlorophyll “a” and Chlorophyll “b”

Under irrigation treatment, chlorophyll “a” was retained in most of the genotypes, while it was only significantly increased in CH 24/07 and significantly decreased in D-09027. Generally, Chlorophyll “a,” under heat stress, decreased significantly in most of the genotypes, while increased significantly in CMC 211S and Bhakhar-2011. No change was observed in D-09013 genotype. Under combined stress treatment, significant increase was observed in same genotypes as under heat stress, while in three genotypes (D-09013, D-09027, and NIFA-2), it remained unchanged despite leftover genotypes showing significant decreased chlorophyll “a” content (Supplementary Figure 8).

Under irrigation, chlorophyll “b” content was found significantly increased in CH104/06 and CMC211, while significant decrease was observed in D-09013 and D-09027. It retained in all others as compared to control. In case of heat stress chlorophyll “b,” contents significantly decreased in most of genotypes, while a significant increase was observed in 3 genotypes (BRC-390, CMC 211S, and Bhakhar-2011). No change was found in CH104/06 and NIFA-2. Under combined stress, significantly increased chlorophyll “b” content was observed in 3 genotypes (CH104/06, TGX 225, and Bhakhar-2011), while three genotypes (D-09013, CH 24/07, and BRC-390) depicted significantly decreased contents, but in left over, it was retained compared with that of non-stress conditions (Supplementary Figure 8).

#### Total Chlorophyll and Carotenoid Content

As compared to non-stress condition, total chlorophyll content was generally retained by most of genotypes under irrigation treatment, while a significant increase was observed in CMC 211S. Genotypes D-09013 and D-09027 depicted significantly decreased contents. Under heat stress, a significant increase was observed in CMC 211S and Bhakhar-2011, while retained in CH104/06, BRC-390 and NIFA-2. It decreased significantly in all others compared to that of control. Under combined heat stress and irrigation treatment, most of the genotypes depicted significant decreased content, while a significant increase was found in CH104/06 and Bhakhar-2011. It was retained in D-09027 and TGX 225 compared to control conditions (Supplementary Figure 8).

Carotenoid content was non-significant in all genotypes except in CH104/06 and CMC 211S in which a significant increase was found. In case of heat stress, most of the genotypes depicted significantly decreased carotenoid content, while it significantly increased in two genotypes (CMC 211S, Bhakhar-2011) and it was retained in another two (BRC-390 and NIFA-2) compared with the control conditions. Under combined stress conditions, a significant increase in carotenoid content was observed in D-09027, CH104/06, NIFA-2, and Bhakhar-2011 while all others showed no change (Supplementary Figure 8).

#### Yield Evaluation

Yield performance was tested across the country, and the consolidated results of yield trial is presented in Table 2. On the basis of average yield performance across all locations, genotypes were grouped into three categories, i.e., high, medium, and low. High class contained 4 genotypes, namely, D-09013, D-09027, CH 24/07, and CH 104/06 with average yield values 1,169, 1,146, 1,211, and 1,285 (kg/ha), respectively, percentage increase for above genotypes from Bhakhar-2011 was observed to be 30.2, 27, 0.6, 34.8, and 43%, respectively, while percentage increase from Bb 2008 was 22.4, 20, 26.8, and 34.6, respectively. The medium group contained 5 genotypes, namely, TGX 225, BRC-390, NIFA-2, Pb-2008 (check), and Bhakhar-2011(check) with values 962, 951, 867, 898, and 955 (kg/ha), respectively, the percentage increase of these genotypes from Bhakhar-2011 was 7.2, 5.9, −3.5, 0, and 6.4%, respectively, and the percentage increase when observed against Pb-2008 for these genotypes were.8, −0.4, −9.2, 0, and −5.9%, respectively. Only one genotype CMC 211S with average yield value 323 kg/ha and percentage decrease from Bhakhar-2011 and Pb-2008 was −0.64 and −66.2%, respectively (Table 2).

**Table 2:** Consolidated yield data of Desi chickpea across 8 locations.

	**Entry name**	**Source**	Locations*								**MEAN**	**% increase from Pb2008**	**% increase from BK-2011**
			**1**	**2**	**3**	**4**	**5**	**6**	**7**	**8**	**Kg/ha**		
1	D-09013	AARI, Faisalabad	639	1,607	1,854	419	580	1,116	1,806	1,331	1,169 High	22.4	30.2
2	D-09027	AARI, Faisalabad	1,053	1,374	1,217	463	441	1,264	1,922	1,435	1,146 High	20.0	27.6
3	CH 24/07	NIAB, Faisalabad	858	1,733	1,639	366	666	1,134	1,921	1,369	1,211 High	26.8	34.8
4	CH 104/06	NIAB, Faisalabad	914	1,512	2,280	468	543	852	2,338	1,376	1,285 High	34.6	43.1
5	TGX 225	AZRI, Bhakhar	312	1,421	1,403	389	465	634	1,806	1,268	962 Medium	0.8	7.2
6	BRC-390	RARI, Bahawalpur	403	1,217	1,259	424	551	796	2,083	876	951 Medium	−0.4	5.9
7	CMC211S	NARC, Islamabad	77	381	161	370	42	884	185	483	323 Low	−66.2	−64.0
8	NIFA-2	NIFA, Peshawar	504	861	1,014	396	225	1,472	1,805	659	867 Medium	−9.2	−3.5
9	PB-2008	Check	427	837	1,352	491	632	898	1,921	1,083	955 Medium	0.0	6.4
10	Bhakhar-2011	Check	190	873	1,616	377	552	884	1,644	1,050	898 Medium	−5.9	0.0
Location Means			601	1,228	1,370	418	486	878	1,754	1,088			

*1 = NIAB, Faisalabad, 2 = PRI, AARI, Faisalabad, 3 = GBRS, Kalurkot, 4 = BARS, Fateh Jang, 5 = AZRI, Bhakkar, 6 = QAARI, Larkana, 7 = RARI, Bahawalpur, 8 = AZRI, D. I. Khan.*

*Coefficient of variation= 14.93% Genotypes (G), Location (L) and G x L interactions are highly significant (P < 0.01)*.

#### Cluster Analysis

**Figure 3:**
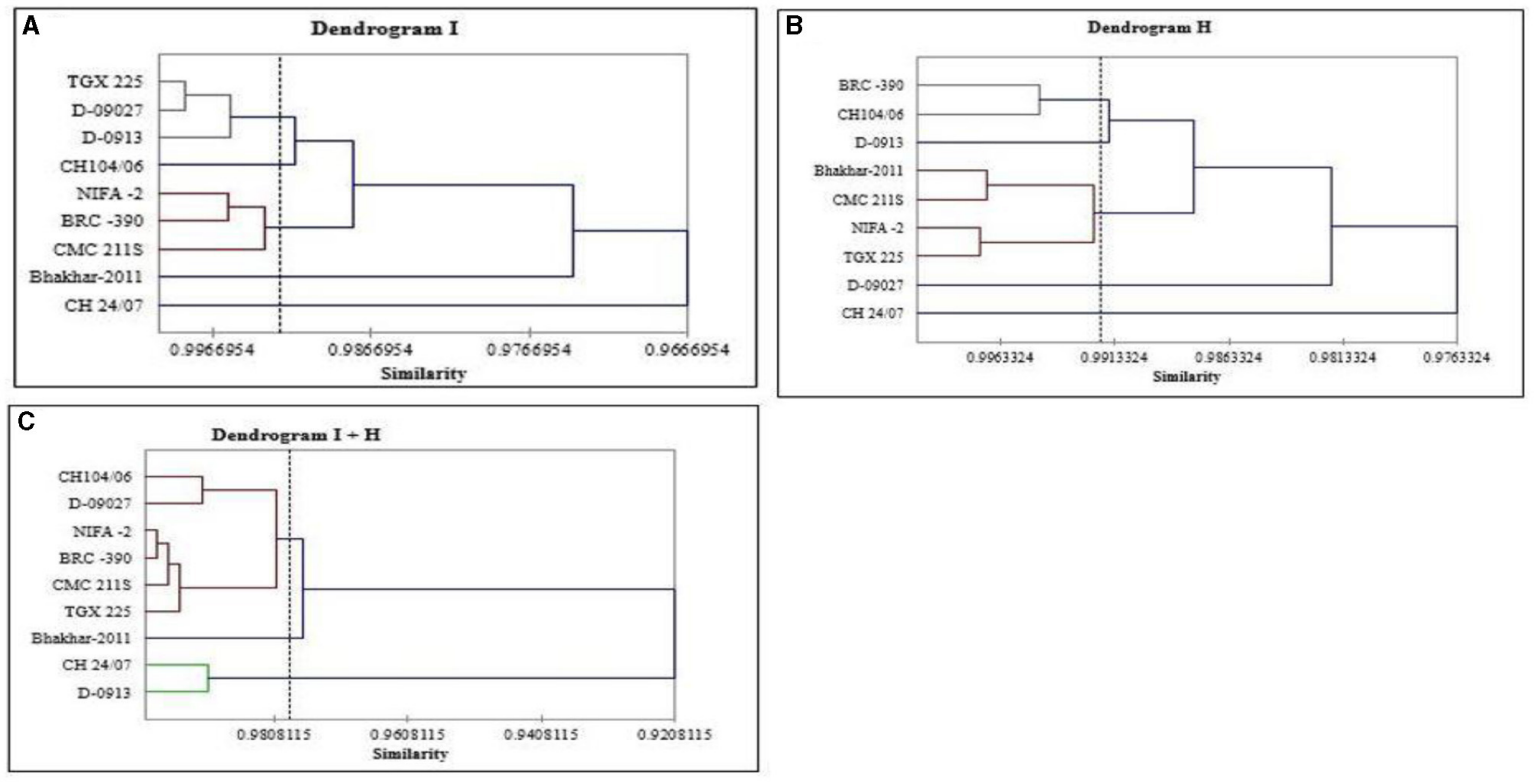
Tree diagram based on morphophysiological and biochemical traits for different chickpea genotypes under **(A)** irrigation, **(B)** heat, and **(C)** combined stress conditions.

For all genotypes, a tree diagram was constructed through hierarchical cluster analysis by using morpho-physiological and biochemical data under irrigation, heat, and combined treatment. In case of irrigation treatment, cluster analysis categorized nine genotypes into five clusters as shown in Table 3 and Figure 3. Three genotypes D-09013, D-09027, and TGX 225 were grouped into Cluster I; Cluster II contained only one genotype CH 24/07; CH104/06 was grouped into cluster III, while 3 genotypes, i.e., BRC−390, CMC 211S, and NIFA−2 were placed into cluster IV and cluster V contained Bhakhar-2011. In case of heat stress, genotypes were grouped into five clusters, genotype D-09013 was placed into cluster I, D-09027 grouped into cluster II, CH 24/07 categorized into cluster III, and Cluster IV contained CH104/06 and BRC−390, while cluster V contained maximum number of genotypes, namely, Bhakhar-2011, TGX 225, CMC 211S, and NIFA−2 (Table 3). Under combined stress, genotypes were grouped into 3 clusters, cluster I contained the genotypes D-09013 and CH 24/07, seven genotypes were grouped into cluster II, namely, D-09027, CH104/06, TGX 225, BRC−390, CMC 211S, and NIFA−2. Cluster III contained only one genotype Bhakhar-2011 (Table 3).

**Table 3:** Classification of chickpea genotypes in different clusters based on morphophysiological and biochemical traits under irrigation, heat, and combined stress treatments.

**Clusters**	**Irrigation treatment**	**Clusters**	**Heat stress**	**Clusters**	**Combined stress**
I	D-09013, D-09027, TGX 225	I	D-09013	I	D-09013, CH 24/07
II	CH 24/07	II	D-09027	II	D-09027, CH104/06, TGX 225, BRC−390, CMC 211S, NIFA−2
III	CH104/06	III	CH 24/07	III	Bhakhar-2011
IV	BRC−390, CMC 211S, NIFA−2	IV	CH104/06, BRC−390		
V	Bhakhar-2011	V	Bhakhar-2011, TGX 225, CMC 211S, NIFA−2,		

#### Correlation Analysis

**Figure 4:**
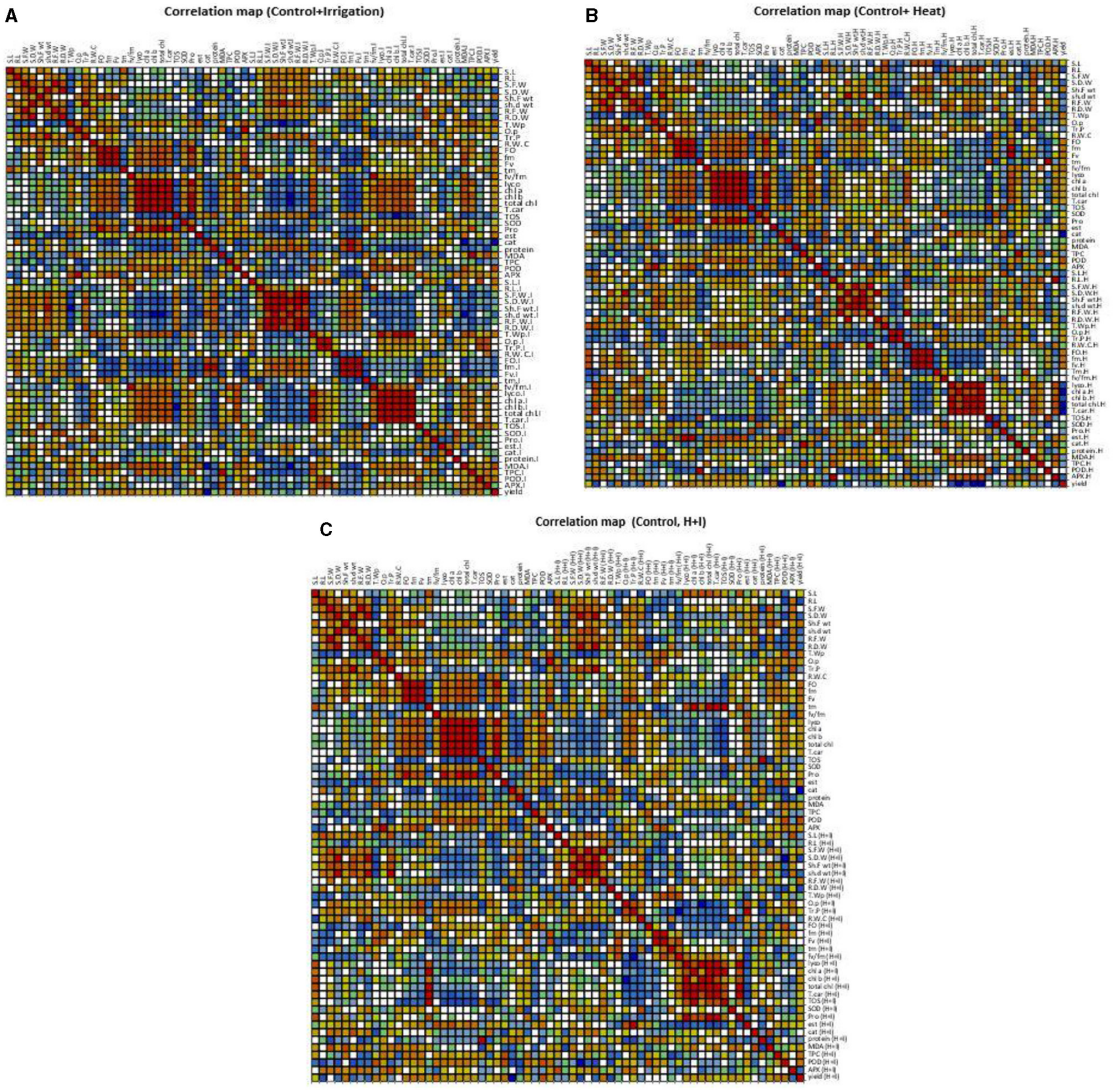
Correlation matrix showing Pearson's correlation among morphophysiological and biochemical traits in chickpea genotypes under Irrigation and non-stress conditions **(A)**. Correlation matrix showing Pearson's correlation among morphophysiological and biochemical traits in chickpea genotypes under Heat and non-stress conditions **(B)**. Correlation matrix showing Pearson's correlation among morphophysiological and biochemical traits in chickpea genotypes under Combined stress and non-stress conditions **(C)**.

Correlation (Pearson) for all morphological and physio biochemical traits under control and irrigation treatment was carried out using XL-STAT XLSTAT 2014.5.03, with 95% confidence interval (Figure 4A and Supplementary Table 2).

Under control conditions, shoot length (S.L) was positively correlated only with time of achieving maximum fluorescence yield (Tm) under control, and no correlation with any parameter was observed in S.L under irrigation treatment. Despite this, root length under control was negatively correlated with total water potential (T. wp) under control which showed that low water potential caused an increased root length, and R.L under irrigation treatment showed a positive correlation with the ratio of variable chlorophyll fluorescence and minimal chlorophyll fluorescence *Fv*/*Fm*, while it negatively correlated with protein content and Tm under irrigation treatment. Seedling fresh weight (S.F.W) under control showed a positive correlation only with root fresh weight under control, which depicted that an increase in root growth had significant effects on plant biomass and growth, while S.F.W under irrigation treatment depicted a positive correlation with shoot dry weight (sh.d wt) under control, seedling dry weight under irrigation (S.D.W.I), shoot fresh weight (Sh.F wt.I), root fresh weight (R.F.W.I), and root dry weight (R.D.W.I) under irrigation treatment. Total water potential (T.W.P) under control negatively correlated with root length (R.L), seedling dry weight (S.D.W) and shoot dry weight (sh. d wt), seedling fresh weight under control (S.F.W) and seedling fresh weight under irrigation (S.F.W.I), seedling dry weight under irrigation (S.D.W.I), shoot fresh weight under irrigation (Sh.F w.I), shoot dry weight under irrigation (Sh.d w.I), and root fresh weight under irrigation (R.F.W.I). In the case of pigments, lycopene (lyco) under control showed a positive correlation with total Chlorophyll, chlorophyll a (chl a), chlorophyll b (chl b), carotenoids (T.car) under control, similarly chl a depicted positive correlation with total chlorophyll and T.car under control. It was noticed that total chlorophyll and chl b had a negative association with TOS under control, so low oxidant status played important role in high pigment contents of plants to cope with different stresses. Chlorophyll fluorescence under irrigation treatment showed a positive association with catalase activity and protein contents under control, while it negatively correlated with TOS and MDA content under irrigation. SOD.I under irrigation treatment had a positive correlation with total soluble protein content plant under irrigation treatment (Figure 4A).

Correlation (Pearson) for all morphological and physio biochemical traits under control and heat stress was carried out using XL-STAT XLSTAT 2014.5.03, with 95% confidence interval (Figure 4B and Supplementary Table 2). Values in bold form are different from 0 with a significance level alpha = 0.05 bold form values with negative sign showed a negative correlation. Root length under heat stress had a positive correlation with total phenolic content under heat stress, it means phenols play an important role in root growth under heat stress, and seedling fresh weight showed a positive correlation with root, shoot fresh weight, and shoot dry weight under heat stress. The ratio of variable chlorophyll fluorescence and minimal chlorophyll fluorescence *Fv*/*Fm Fv*/*Fm* under heat stress showed a positive correlation with SOD activity which play a role as an antioxidant to scavenge reactive oxygen species generated in plant under heat stress conditions, so increased SOD activity caused an increase in ratio *Fv*/*Fm* in tolerant genotypes. Lycopene content in plants under heat stress showed a positive association with chlorophyll a, total chlorophyll, and total carotenoids under heat stress. In addition, it also acts as an antioxidant. TOS under heat stress showed a negative correlation with lycopene, chlorophyll a, total chlorophyll, total carotenoids, and SOD. These indices could be good markers to check tolerance in plants under heat stress. POD under heat stress had a positive correlation with TOS under heat stress, while ascorbate APX under heat stress showed a positive correlation with lycopene and MDA under heat stress conditions. Lycopene content positively correlates with all types of pigments measure under heat stress and negatively correlates with esterase activity under heat stress, chlorophyll positively correlates with all other types of pigment contents measured under heat stress, so these attributes could serve as stress markers.

Correlation (Pearson) for all morphological and physio biochemical traits under control and combined stress was carried out using XL-STAT XLSTAT 2014.5.03, with 95% confidence interval (Figure 4C and Supplementary Table 2). Values in bold form are different from 0 with a significance level alpha = 0.05 bold form values with negative sign showed negative correlation. Among morphological parameters, Seedling length under combined stress condition depicted positive correlation with APX activity under combined stress exposure, root length under combined stress [R.L (H+I)] showed negative association with MDA (H+I). S.F.W (H+I) had positive correlation with R.F.W (H+I) and negative association with *Fv*/*Fm* (H+I). S.F.W (H+I) showed positive correlation with Sh.F wt (H+I) and R.F.W (H+I). S.D.W (H+I) showed positive association with sh.d wt (H+I) and negative with POD (H+I). Sh.F wt (H+I) had positive correlation with sh.d wt (H+I), S.F.W (H+I) and negatively correlate with *Fv*/*Fm* (H+I). Sh.d wt (H+I) showed positive correlation with S.D.W (H+I), Sh.F wt (H+I), and negatively correlated with POD (H+I).

Among physiological traits, T.Wp (H+I) showed positive correlation with Fv (H+I), tm (H+I), and *Fv/Fm* (H+I) and negatively associated with Tr.P (H+I). O.p (H+I) had positive correlation with Tr.P (H+I), and negatively correlated with lyco (H+I), total chl (H+I), and total chl (H+I). Among biochemical traits protease enzyme activity positively correlates with all pigments under heat stress, esterase activity under heat stress negatively correlated with *Fv*/*Fm* (H+I), lyco (H+I), and T.car (H+I), catalase activity positively correlates with MDA (H+I).

### Principal Component Biplot Analysis

For a better understanding of the relationship among genotypes and to extract the important and useful information present in the data matrix, principle component analysis was performed for all genotypes and 33 traits under investigation in each treatment. Principle component analysis also minimized the number of traits which explains the maximum percentage of variability present in the data matrix. The eigenvalue is imperative to decide which principal components are important for further study. Approximately, 10% of the total variation explains by Principle components with more than 1 Eigenvalue (Brejda et al., 2000). The higher Eigen values were reflected as best representative of system aspects in principal components (Kumar et al., 2019). Under Irrigation 8, Principle components (PCs) depicted more than 1.0 Eigenvalue and explained 100% variability. The first two components were most influential, PC-I and PC-II individually contributed 34.319 and 17.73% of total variability, respectively, while cumulatively both contributed 52.051% of the variability. In the case of heat stress, eight PCs depicted an Eigenvalue of more than 1 and revealed 100% of cumulative variability. Maximum variability was explained by the first two PCs, individually PC-I and PC-II contributed 29.68 and 17.40% of total variability, respectively, and cumulatively they contributed 46.94% of total variability. Under combined stress of heat and irrigation out of 8 PCs, showed Eigenvalue more than 1.0 and contributed 100% of cumulative variability. The first two components, PC-I and PC-II, contributed individually, 25.89 and 22.18% of total variability, respectively, and cumulatively they contributed 48.075% of the total variability (Supplementary Table 1).

Out of 33 traits under investigation in irrigation treatment, only 13 traits showed positive factor loading in PC-I, while seedling dry weight (0.859), seedling fresh weight (0.831) and shoot fresh (0.813), shoot dry weight (0.838), and root dry weight had the greatest effect in PC-I. In PC-II, 11 traits exhibited positive factor loading with tm and catalase having the greatest effect. Under heat stress, PC-I and PCII revealed 47.09% of cumulative variability. In PC-I, out of 33 parameters under study, 23 traits represented positive factor loading, while total carotenoids (0.935), lycopene (0.887), total chlorophyll (0.861), and chlorophyll a (0.807) depicted the greatest contribution. In PC-II, out of 33 traits, 13 traits represented positive factor loading, while maximum contribution was given by shoot fresh weight (0.817). In combined treatment, out of 33 traits, 14 traits depicted positive factor loading in PC-I, however, the maximum contribution was given by lycopene (0.872), total chl (0.86), and total car (0.814). In PC-II, 20 traits exhibited positive factor loading. However, lycopene (0.96), total chlorophyll (0.92) and total carotenoids (0.90) had the greatest effect (Supplementary Table 1).

**Figure 5:**
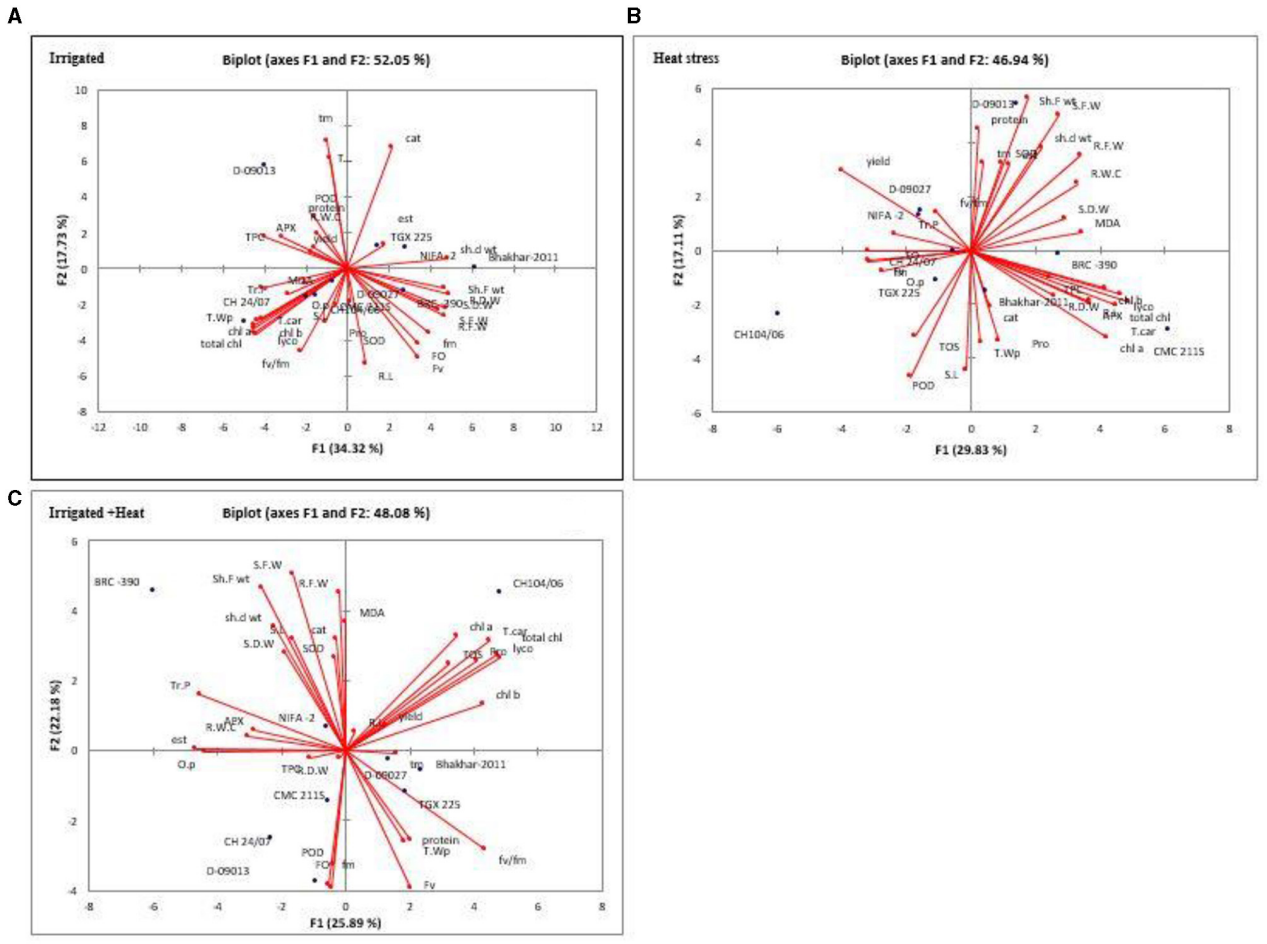
Biplots of first two principal components (PC-I and PC-II) for irrigated **(A)**, heat **(B)**, and combined (H+I) effect **(C)**. S.L, Shoot Length; R.L, Root Length; S.F.W, Seedling Fresh Weight; Sh. F Wt, Shoot Fresh Weight; Sh.D Wt, Shoot Dry Weight; R.F.W, Root Fresh Weight; R.D.W, Root Dry Weight; T. W P, Total Water Potential; O.P, Osmotic Potential; Tr.P, Turgor Potential; R.W.C, Relative Water Content; Fo, Minimal Fluorescence; Fm, Maximal Fluorescence; Fv, Variable Fluorescence; Fv/Fm, ratio of variable chlorophyll fluorescence and minimal chlorophyll fluorescence; Lyco, Lycopene; Chla, Chlorophyll A; Chlb, Chlorophyll B; Total Chl, Total Chlorophyll; T.Car, Total Carotenoids; TOS, Total Oxidant Status; SOD, Superoxide Dismutase; CAT, Catalase; TSP, Total Soluble Protein; MDA, Malondialdehyde; TPC, Total Phenolic Contents; POD, Peroxidase; APX, Ascorbate peroxidase; Tm, time of achieving maximum fluorescence yield.

For more reliable identification of genotype with maximum value for one or more traits, genotype by trait (GT) biplots were constructed for PC-I and PC-II for all genotype and all traits under all treatments (Figure 5). It exhibited the trait profile of a genotype (Turner, 2016). In biplots to understand the interrelationship among genotypes and traits, vector lines were drawn from the origin of the biplots. Genotypic performance (how it differs from average genotype) can be estimated by the distance of genotype from the origin of the biplot, distant genotypes could have maximum values for one or more traits. Pearson correlation between traits were indicated by cosine angle between two traits, i.e., no correlation = angle of 90°: positive correlation = <90°: negative correlation = >90° (Argentel-Martínez et al., 2019). Under irrigation treatment genotypes, D-09013 was a distant genotype and positive correlation was observed among POD, yield, RWC, TOS, APX, TSP, and TPC. In case of heat stress, genotypes D-09013 with positive correlation [seedling dry weight, yield, superoxide dismutase (TOS), TSP, shoot fresh weight, seedling fresh weight, shoot dry weight, root fresh weight, esterase], and CH 104/06 with positive correlation among osmotic potential, TOS, shoot length, and peroxidase activity was distant. It was observed that under combined treatment CH 104/06 with positive correlation among chlorophyll a, chlorophyll b total chlorophyll, total carotenoids, protease, TOS, yield, lycopene, root length, and BRC-390 were distant with positive correlation among SOD, shoot length (S.L), catalase (cat), shoot fresh weight (sht. F wt), shoot dry weight (sh. d wt), seedling fresh weight (S.F.W) and seedling dry weight (S.D.W) (Supplementary Table 1).

## Discussion

Abiotic stresses are mostly interlinked, either independently or in the association, causing morphological, physiological, biochemical, and molecular modification that negatively affects crop development, crop efficiency, and finally, yield (Bita and Gerats, 2013). The prevalence of inconsistent rainfall patterns and temperature extremes (heat and cold) are anticipated to increase in the near future owing to climate change, thereby compelling the development of stress-tolerant and climate-smart chickpea cultivars (Pereira, 2016; Raza et al., 2019; Rani et al., 2020). It has been noticed that heat and low moisture affects the early morphological stages of chickpea plant indirectly yield affected badly (Wahid et al., 2007).

The main objectives of this study were to identify the irrigation responsive, tolerant genotypes for heat stress, and best performing genotypes for a combination of both these conditions at the seedling along with an assessment of yield potential. Present findings revealed that at 100% field capacity a substantial increase in shoot length, shoot fresh weight, shoot dry weight, root length, root fresh, and dry weight was noticed in some genotypes. Previously, it was reported that, in chickpeas, at 100% field capacity, plant biomass was increased because plant growth response was positively correlated with the availability of soil moisture content, it was also reported that there was a positive association between high early vigor and plant yield (Hosseini et al., 2009). Previously it was reported that irrigation supplementation of chickpea cultivars revealed positive effects on micro-organisms activity, phosphate solubilization, and crop growth (Ilhe et al., 2009). Another report suggested that irrigation application significantly increased number of nodules and grain yield in chickpea (Patel et al., 2014). In the case of heat stress, the shoot length was decreased in some genotypes, while in CH104/ 06 and D-09013, there was a non-significant reduction in shoot and root length, seedling dry weight, shoot fresh and weight, root dry weight, relative water content was observed, so these genotypes could be tolerant under heat stress. In general, heat stress reduced plant growth, specially shoot or root length and shoot or root dry weight in chickpea, heat tolerant genotypes retained these attributes (Thorsted et al., 2006; Kiran and Chimmad, 2018). Similar results were reported in chickpea tolerant genotypes Raj-4037 and PBW590 under heat stress condition (Chaudhary et al., 2020). In other reports, it was observed that in maize, sorghum, and soybean under low soil moisture content diminishes, plant height, plant leaf and growth, dry matter, and eventually yield in susceptible genotypes (Ghosh et al., 2000; Khan et al., 2015). It was observed that in the case of heat stress exposure in chickpea the morpho-physiological parameters varied significantly, the same behavior was observed in the present study (Wahid and Close, 2007; Soureshjani et al., 2021). Under heat stress with supplementation of water at 100% field capacity shoot length, seedling fresh weight, seedling dry weight, root length, seedling fresh weight, seedling dry weight, shoot fresh and dry weight was retained in CH104/06 and significantly increased root fresh weight was observed, so this could be the best performing genotype, as it also showed 43% yield increment over check. Previously, it was suggested that, in chickpea, a positive association exists between crop establishment and yield with high early vigor under heat stress with supplementation of irrigation (Yadav et al., 2005). For horticultural crops, plant water status is the major factor affecting the yield and quality. Recently, to determine plant water status water potential is being increasingly used (Jones, 1990). Previously, it was reported that additive gene action involved in controlling root length, root density, and root dry weight (Devasirvatham and Tan, 2018).

The study suggested additive gene action control root length density and root dry weight (Khamssi et al., 2010). Transcriptional factors (TFs) play a vital role in modulating cellular response under stress situations by activating the transcription of target genes. In the regulation of transcriptional reprogramming linked with plant stress reactions, the WRKY gene family has been proposed to play imperative roles (Chen et al., 2012). Seven genes were identified and analyzed under heat stress in chickpea genome, i.e., *ARP6* (actin-related protein), two *SEF* (serrated leaf and early flowering), three *H2AZs* (histone 2A variant-Z,a thermosensor in plants) and *PIE1* (photoperiod independent early flowering 1) (Chidambaranathan et al., 2016).

Under irrigation, for total water potential significantly decreased in all genotypes. In addition, differences were observed for all genotypes. Under heat stress, water potential significantly increased in D-09013 and D-09027, these genotypes depicted tolerant behavior under heat stress because increased water potential allows water movement from outside into cells to maintain its turgidity, these genotypes also revealed high yield potential with 30 and 27.6% yield increment over check. It was studied in maize that, in susceptible genotypes, a decreased water potential was associated with stomatal closure and causing a decrease in carbon assimilation and photosynthesis (Kim et al., 2018; Sattar et al., 2020). In the case of combined heat stress, a non-significant change was observed for all genotypes except BRC-390 and Bhakhar-2011 which showed a significant decrease in total water potential. Under low moisture content in the soil and high atmospheric temperature, a decrease in transpiration rate and leaf water potential was observed which consequently increase canopy temperature cause susceptibility (Fahad et al., 2017). It can be suggested, that under a combination of both heat and water supplementation non-significant results were observed, maybe water application dissipate the effect of heat stress by increasing transpiration rate and causing cooling effects in plant, and in this way, it maintains water potential in leaves. It was reported previously in potato, low leaf water potential, results in decreasing leaf turgor potential and consequently, leaf wilting occurs under low water availability and high temperature (Handayani and Watanabe, 2020).

Osmotic regulation plays a vital role in plant adaptation to abiotic stresses (Turner, 2016). Osmotic water potential was significantly decreased in most genotypes, while an increase was observed in CH104/06 and CMC 211S under irrigation. Osmotic water potential significantly decreased in D-09013, Bhakhar-2011, and BRC-390, which meant that these genotypes could have better adaptability while under combined stress, it decreased in all. The previous finding revealed that in plants, under abiotic stresses (heat and drought), a decrease in osmotic potential was observed by accumulating, osmotically active, low molecular weight compounds (osmolytes) such as glycine betaine (GB), proline, and reduced glutathione (RG) in chickpea tolerant genotypes (Argentel-Martínez et al., 2019; Makonya, 2020). The pressure exerted against the plant cell wall by protoplast is termed as turgor potential. High turgor pressure is important to give plants their structure, rigidity, mediate stomatal opening and closing, etc. (Nongpiur et al., 2020). Under Irrigation, turgor potential was significantly increased in D-0913, CH24/07, CMC 211S, and CH104/06, while under heat stress, a significant increase was observed for most of the genotypes. So, genotypes depicted with high turgor potential could be tolerant under heat stress, i.e., CH104/06 and D-09027. Under heat, the loss of turgor pressure in the cells hindered turgor-dependent processes such as cell expansion, which affects the growth and development of the whole plant (Arve et al., 2011).

In susceptible genotypes, the water content in the cells is quickly decreased to the extent where the osmotic pressure is reduced and the cells lose turgor pressure and wilting occurs (Luan, 2002). The *Fv/Fm* ratio is an appropriate index for evaluating PSII in chickpea exposed to environmental stresses such as heat and water stress (Hosseinzadeh et al., 2018). For early stress detection in plants, chlorophyll fluorescence parameter *Fv*/*Fm* has been widely used because it revealed the maximum quantum efficiency of photosystem II (PSII) photochemistry (Sharma et al., 2015). Under irrigation, non-significant changes were observed for *Fv*/*Fm* for all genotypes, the same results were found in previous research on bread wheat under well-watered and water-limited conditions (Del Pozo et al., 2020). Earlier, a study revealed that two heat tolerant genotypes of chickpea depicted *Fv*/*Fm* value ranging 0.83–0.85, so CH104/06 could be a heat tolerant genotype because it depicted *Fv*/*Fm* 0.83 value (Makonya et al., 2019). Relative water content (RWC) of all genotypes under irrigation increased significantly, while it retained in heat and combined stress situation. Earlier reports stated that heat stress negatively affected relative water content in chickpea (Kumari et al., 2018). Similar findings were also observed in mung bean (*Vigna radiata*) under high temperature stress (Kumar et al., 2011).

Due to unpredicted climate changes, pulses have become more prone to oxidative damage by excessive production of toxic ROS such as hydrogen peroxide, superoxide and hydroxyl radicals (Sultana et al., 2014). To combat ROS generated under abiotic stresses, specialized enzymatic antioxidants, i.e., SOD, POD, CAT, and APX get activated and act as the first line of defense to detoxify the effects of ROS (Tang et al., 2019; Jameel et al., 2021). SOD convert ${\text{O}}_{2}^{∙-}$ into H_2_O_2_ for further reduction to water by CAT and POD (He et al., 2018). Increased enzymatic antioxidants SOD and POD activity under irrigation were found in D-09027, APX and catalase increased in D-09013, genotypes having high enzymatic antioxidants at 100% field capacity could be the best performer in irrigated conditions. Contrary In Osage orange (*Maclura pomifera*), it was reported that at 100% field capacity, SOD activity was non-significant, rather their activity increased with a decrease in moisture level (Khaleghi et al., 2019). However, under heat stress, a significantly increased SOD was observed in Bhakhar-2011 and D-09027, while catalase activity increased in Bhakhar-2011 and retained in D-09027. Similarly, in green gram, elevated SOD and catalase activity under heat stress was investigated (Rakavi and Sritharan, 2019). Under exposure to heat stress, increased POD and APX activity was observed in D-09013 and D-09027. These genotypes showed heat tolerance as they depicted increased enzymatic antioxidant potential. Similar findings were reported in chickpea tolerant genotype RSG 888 under heat stress in which increased antioxidants was observed (Kumar et al., 2017; Shafiq et al., 2021).

It has been reported that heat tolerance in chickpea is strongly associated with an increase in antioxidant enzyme activity and induction of heat shock proteins (Parankusam et al., 2017; Tao et al., 2020; Wang et al., 2021). Under combined stress, a significant increase in POD was found in D-09027 and CH24/07, while SOD activity remained similar as in control. In maize and wheat, it was reported that heat stress could be considerably decreased by irrigation due to surface cooling occurs as a result of certain physiological and biochemical changes (Siebert et al., 2017). Phenolic compounds are an important class of plant secondary metabolites, possess redox properties, and part of non-enzymatic antioxidants (Giannakoula et al., 2021). Under exposure to abiotic stress conditions, plants exhibit increased production of polyphenols such as flavonoids, phenolic acids, which help the plant to handle environmental constraints (Sharma et al., 2019). Under irrigation, total phenolic contents (TPC) were increased in D-09013, D-09027, CH104/06, and Bhakhar-2011. These genotypes could have great potential to grow under irrigation treatment. Earlier, it was noticed that an increase in TPC were observed in Bhakhar-2011 under well-watered and low water stress conditions, which is in accordance with the present study (Farooq et al., 2018). The previous report suggested that plants grown in soil showed less phenolic contents as compared to plants grown aeroponic system which showed higher phenolic contents and yield in vegetables (Chandra et al., 2014).

Heat stress induced significantly decreased TPC in all genotypes as compared to non-stress conditions. The previous investigation on soybean (*Glycine max*) was in agreement with present findings, as in soybean TPC decreased significantly (18%) under heat stress (38/28°C) (Hasanuzzaman et al., 2013). TPC was decreased under combined stress in D-09027, CH 24/07, CMC 211S, and NIFA-2, while in remaining genotypes combined stress showed no effect on TPC. It was clear from previous investigations on chickpea that the accumulation of phenolics and flavonoids induces heat tolerance in ICC 1205 and is an important biochemical marker for heat tolerance studies (Pareek et al., 2019).

Another biochemical reaction of the plants, under abiotic stresses (heat, salinity, drought, flooding, etc.) involves the modulation of different hydrolytic enzymes such as esterases, and proteases (Ehsanpour and Amini, 2003). Among all the genotypes, esterase activity under irrigation, heat, and combined stress decreased significantly in Bhakhar-2011, while in some genotypes it increased and in others, retained. Earlier, it was observed that a marked increase in esterase activity, under low moisture in cotton (*Gossypium hirsutum*) was observed in tolerant genotypes (Prajapat et al., 2018). Under irrigation and heat stress, protease activity was decreased in all genotypes, while the least protease activity observed in Bhakhar-2011 followed by D-09013. However, in combined, only CH104/06 showed significant increase protease activity and was retained in leftovers. It was previously reported that in chickpea under low water proteases have indispensable roles in plants by maintaining protein quality control and degrading unstructured peptides and specific sets of damaged proteins (Vessal et al., 2020). A previous report stated that elevated protease activity is the indication of more proteolytic enzyme activation in heat susceptible wheat genotypes, it also showed positive correlation with heat susceptibility index (HIS) (Hameed et al., 2012).

Under irrigation, heat, and combined stress TOS was significantly increased in the majority of genotypes, while it decreased significantly in BRC-390 and was retained in some genotypes. The previous investigation on chickpea indicated that under heat and low moisture stress exposure oxidant status of sensitive genotypes increased due to oxidative stress and hence, plant yield was badly affected (Kaloki et al., 2019).

Generally, membrane lipid peroxidation in plants is detected by assessing MDA. MDA is an extensively used marker of oxidative lipid damages caused by abiotic stress. There was a positive correlation between MDA concentration and lipid peroxidation and it can deteriorate cell wall integrity (Kong et al., 2016; Rani et al., 2020). Heat stress caused MDA contents to decrease significantly in D-09013 and CH104/06, so these genotypes could have better adaptation capacity under heat stress. These results were in accordance with a previous report, in which heat tolerant chickpea genotypes had a lower accumulation of MDA as compared to sensitive genotypes (Chaudhary et al., 2020).

It was studied earlier in chickpea that at 34 and 36°C, a dramatic increase in MDA was noticed showing higher oxidative damages (Yadav et al., 2021). For cell proper functions, protein molecules play a crucial role (Sara et al., 2012). It was studied that heat stress caused, denaturation of existing proteins and misfolding of newly synthesized proteins (Bita and Gerats, 2013). Under irrigation and heat and combined stress, total soluble proteins (TSP) were significantly increased in D-09013, this genotype may have better adaptability under changing environmental conditions, with 30.2% yield increment over check which implies that high soluble proteins under heat stress may repair heat induced membrane injuries in this genotype. Heat shock genes encoded different heat shock proteins (HSPs) under high temperature stress, which stored and protected cells by acting as molecular cheprons (Huang and Xu, 2008). Identification of 22 Hsfs genes in both desi and kabuli genome has provided valuable knowledge about thermos tolerance (Chidambaranathan et al., 2018). It has been studied that *CarHsfA2, A6*, and *B2* were unregulated in heat stressed chickpea at 15-day-old seedlings and during pod filling stage. Therefore, in the above mentioned genotype, increased proteins may be associated with the upregulation of heat shock proteins (Chidambaranathan et al., 2018). Several imperative candidate genes, *viz*. *Ca_23016, Ca_25811, Ca_09743*, were identified in various reproductive and vegetative tissues subjected to heat stress, which contributed to heat-stress tolerance (Agarwal et al., 2016).

It was reported that low water and high temperature caused a reduction in protein production, which may be due to multifarious factors involvement. Thus causing a reduction in plant growth and crop yield in sensitive genotypes chickpea genotypes (El-Beltagi et al., 2020). The findings were in agreement with previous results in alfalfa (*Medicago sativa*), which depicted that maximum accumulation of soluble protein content was observed in thermotolerant genotypes (Wassie et al., 2019). However, a decrease in total soluble proteins was observed in the case of strawberry and mulberry under high temperature (Ra and Uj, 1962; Gulen and Eris, 2004).

In plants, photosynthesis plays an imperative role in determining growth and development. Numerous studies exhibited that above the thermal threshold, a decline in photosynthesis rate occurs, which can be detected by assessing photosynthetic pigments in leaves (Zafar et al., 2017; Wittmann et al., 2021). Against environmental stresses, lycopene plays a crucial role in the biosynthesis of carotenoids and act as an antioxidant by scavenging singlet oxygen and quenching peroxyl radicals (Jangid and Dwivedi, 2017). Lycopene was increased significantly in CH104/06 under irrigation, heat stress, and combined stress, so this genotype could be the best performer under all treatments. Heat stress badly affects photosynthesis in plants which leads to a reduction in plant vegetative growth (Marchand et al., 2005). Under heat and combined stress conditions, total chlorophyll, chlorophyll b, and carotenoid were increased significantly in CH104/06, which could be the highest tolerant genotype that showed 43% yield increment over check. Similar results were reported in chickpea heat tolerant genotypes, which depicted high chlorophyll and photochemical efficiency than sensitive genotypes (Kaloki et al., 2019). Thermotolerance can be found in the genotypes depicting high chlorophyll pigments. Here, it was found that chlorophyll contents are more responsible for thermotolerance, which can be used as a biochemical marker for heat tolerance studies in chickpea. Similar results were found in tomato chlorophyll contents under heat stress in tolerant genotypes (Nagarajan and Nagarajan, 2009). In chickpea, genome wide association studies (GWAS) have also been used for unraveling the genetic architecture of various complex desired traits for breeding (Rani et al., 2020). Previously, five significant markers-trait associations (MTAs) were deciphered from 71 chickpea genotypes, for chlorophyll content and cell membrane stability related heat tolerance (Jha et al., 2018).

## Conclusion

To the best of current knowledge, limited research is available in chickpea to understand the effects of irrigation, heat, and combined stress at the seedling stage. Therefore, it is the first report in which a quick screening strategy was designed to screen the climate smart and input responsive genotypes at the early growth stage for the aforementioned stresses. By analyzing morphophysiological and biochemical attributes, stress tolerant genotypes were identified. To validate the results, the field performance of lines was also checked across the country. Among all studied traits, seedling fresh and dry weight, root fresh and dry weight, and among biochemical attributes total carotenoids, esterase and protease activity explained more variability in irrigation treatment, while under heat and combined stress, total carotenoids, total chlorophyll, chlorophyll a, chlorophyll b, lycopene and protease explained more data variability. D-09013 and D-09027 were found to be the best performing genotypes in irrigation treatment, D-09013 and CH104/06 as most heat tolerant, and CH104/06 were the best genotype under combined treatment and these genotypes also depicted high yield potential across multiple locations. The identification of the best performing and tolerant genotypes can further be utilized for breeding climate-smart chickpea genotypes for sustainable production under a changing climate.

## Data Availability Statement

The original contributions presented in the study are included in the article/Supplementary Material, further inquiries can be directed to the corresponding author/s.

## Author Contributions

SJ did the overall execution of the experiment, analytical work, collection of data, organization of resulting data, write up, and revision of manuscript. AH performed the basic idea and planning, designed the experiments, helped the data analysis, wrote, revised, and finalized the manuscript. TS performed the basic idea and planning of the experiments and wrote, revised, and finalized the manuscript. All authors contributed to the article and approved the submitted version.

## Conflict of Interest

The authors declare that the research was conducted in the absence of any commercial or financial relationships that could be construed as a potential conflict of interest.

## Publisher's Note

All claims expressed in this article are solely those of the authors and do not necessarily represent those of their affiliated organizations, or those of the publisher, the editors and the reviewers. Any product that may be evaluated in this article, or claim that may be made by its manufacturer, is not guaranteed or endorsed by the publisher.
