# Supplementary material for: Investigation of Distinctive Morpho-Physio and Biochemical Alterations in Desi Chickpea at Seedling Stage Under Irrigation, Heat, and Combined Stress
Source: Front Plant Sci. 2021 Sep 27;12:692745. doi: 10.3389/fpls.2021.692745 (PMC8503603; doi:10.3389/fpls.2021.692745)
Supplement: Supplementary file 1 [file Data_Sheet_1.docx]

Supplementary material

|  |  |
| --- | --- |
|  |  |

Figure. S1: Impact of Irrigation, Heat and combined (Heat +Irrigation) stress on, shoot length (S.L), root length (R.L) seedling fresh weight (S.F.W) and seedling dry weight (S.D.W) of all genotypes. Data presented as mean ± SE, genotypes with different alphabets are statistically significantly different at P < 0.05.

|  |  |
| --- | --- |
|  |  |
| Figure. S2: Impact of Irrigation, Heat and combined (Heat +Irrigation) stress on, shoot fresh weight, shoot dry weight, root fresh weight and root dry weight of all genotypes. Data presented as mean ± SE, genotypes with different alphabets are statistically significantly different at P < 0.0.5 | |

|  |  |
| --- | --- |
|  |  |
| Figure. S3: Impact of Irrigation, Heat and combined (Heat +Irrigation) stress on, total water potential, osmotic potential, turgor potential and relative water content of all genotypes. Data presented as mean ± SE, genotypes with different alphabets are statistically significantly different at P < 0.05. | |

|  |  |
| --- | --- |
|  |  |
| Figure. S4: Impact of Irrigation, Heat and combined (Heat +Irrigation) stress FO, Fm, FV, and TM of all genotypes. Data presented as mean ± SE, genotypes with different alphabets are statistically significantly different at P < 0.05. | |

|  |  |
| --- | --- |
|  |  |
| Figure. S5: Impact of Irrigation, Heat and combined (Heat +Irrigation) stress on, FV/FM, SOD, POD and CAT of all genotype. Data presented as mean ± SE, genotypes with different alphabets are statistically significantly different at P < 0.05. | |

|  |  |
| --- | --- |
|  |  |
| Figure. S6: Impact of Irrigation, Heat and combined (Heat +Irrigation) stress on, APX, TPC, Esterase and protease of all genotypes. Data presented as mean ± SE, genotypes with different alphabets are statistically significantly different at P < 0.05. | |

|  |  |
| --- | --- |
|  |  |
| Figure. S7: Impact of Irrigation, Heat and combined (Heat +Irrigation) stress on, TOS, MDA, Total soluble proteins and lycopene of all genotypes. Data presented as mean ± SE, genotypes with different alphabets are statistically significantly different at P < 0.05. | |

|  |  |
| --- | --- |
|  |  |
| Figure. S8: Impact of Irrigation, Heat and combined (Heat +Irrigation) stress on, chl a chl b, total,chl, and total carotenoids of all genotypes. Data presented as mean ± SE, genotypes with different alphabets are statistically significantly different at P < 0.05. | |

**Table: S1:** Principal component analysis for morpho-physiological and biochemical parameters under Irrigation, Heat and Combined stress

| **Principal Component Analysis:** | | | | | | | | |
| --- | --- | --- | --- | --- | --- | --- | --- | --- |
| **Irrigated** | **PC-I** | **PC-II** | **PC-III** | **PC-IV** | **PC-V** | **PC-VI** | **PC-VII** | **PC-VIII** |
|  |  |  |  |  |  |  |  |  |
| Eigenvalue | 11.325 | 5.851 | 4.174 | 3.426 | 3.077 | 2.013 | 1.850 | 1.283 |
| Variability (%) | 34.319 | 17.732 | 12.647 | 10.382 | 9.325 | 6.101 | 5.606 | 3.888 |
| Cumulative % | 34.319 | 52.051 | 64.698 | 75.079 | 84.404 | 90.506 | 96.112 | 100.000 |
| **Factor loadings:** | |  |  |  |  |  |  |  |
|  | **PC-I** | **PC-II** | **PC-III** | **PC-IV** | **PC-V** | **PC-VI** | **PC-VII** | **PC-VIII** |
|  |  |  |  |  |  |  |  |  |
| S.L | -0.096 | -0.254 | 0.651 | 0.552 | 0.378 | 0.034 | -0.048 | -0.228 |
| R.L | **0.146** | -0.675 | -0.453 | 0.489 | -0.038 | -0.157 | -0.205 | -0.101 |
| S.F.W | **0.831** | -0.283 | -0.230 | 0.239 | -0.154 | 0.146 | 0.203 | 0.180 |
| S.D.W | **0.859** | -0.176 | 0.012 | 0.206 | -0.249 | 0.313 | -0.075 | 0.150 |
| Sh.F wt | **0.813** | -0.142 | -0.169 | 0.117 | -0.254 | 0.158 | 0.417 | 0.116 |
| sh.d wt | **0.838** | **0.074** | 0.256 | -0.120 | -0.374 | 0.248 | 0.003 | -0.103 |
| R.F.W | **0.816** | -0.331 | -0.248 | 0.281 | -0.110 | 0.137 | 0.113 | 0.200 |
| R.D.W | **0.763** | -0.285 | -0.118 | 0.352 | -0.152 | 0.308 | -0.106 | 0.264 |
| T.Wp | -0.760 | -0.371 | 0.012 | 0.090 | 0.185 | 0.453 | 0.158 | 0.107 |
| O.p | -0.501 | -0.180 | 0.619 | 0.282 | -0.481 | -0.066 | -0.116 | -0.075 |
| Tr.P | -0.357 | -0.107 | 0.648 | 0.276 | -0.547 | -0.171 | -0.158 | -0.103 |
| R.W.C | -0.250 | **0.247** | -0.440 | -0.082 | -0.570 | -0.368 | -0.401 | 0.236 |
| FO | **0.595** | -0.535 | 0.353 | -0.372 | -0.025 | 0.228 | -0.079 | -0.193 |
| fm | **0.692** | -0.454 | 0.417 | -0.346 | -0.047 | 0.075 | -0.077 | -0.086 |
| Fv | **0.601** | -0.629 | 0.316 | -0.331 | -0.025 | -0.070 | -0.040 | -0.165 |
| tm | -0.180 | **0.896** | -0.206 | -0.210 | 0.053 | 0.188 | 0.184 | 0.075 |
| fv/fm | -0.394 | -0.588 | -0.421 | 0.101 | 0.091 | -0.457 | 0.172 | -0.254 |
| lyco | -0.788 | -0.423 | -0.127 | -0.123 | -0.272 | 0.234 | 0.186 | 0.076 |
| chl a | -0.725 | -0.357 | -0.178 | -0.283 | -0.316 | -0.004 | 0.251 | -0.270 |
| chl b | -0.778 | -0.469 | -0.094 | -0.069 | -0.257 | 0.196 | 0.198 | 0.134 |
| total chl | -0.785 | -0.453 | -0.119 | -0.128 | -0.280 | 0.148 | 0.218 | 0.030 |
| T.car | -0.792 | -0.400 | -0.143 | -0.180 | -0.272 | 0.229 | 0.180 | -0.040 |
| TOS | -0.149 | **0.774** | 0.116 | 0.307 | -0.290 | 0.380 | 0.206 | -0.013 |
| SOD | **0.021** | -0.232 | 0.670 | 0.061 | 0.131 | -0.340 | 0.375 | 0.469 |
| Pro | -0.184 | -0.375 | 0.412 | -0.285 | 0.658 | 0.242 | -0.198 | 0.210 |
| est | **0.313** | **0.167** | 0.439 | 0.073 | -0.347 | -0.227 | 0.686 | -0.185 |
| cat | **0.378** | **0.853** | -0.077 | -0.214 | -0.033 | -0.040 | 0.234 | -0.145 |
| protein | -0.284 | **0.146** | 0.619 | -0.481 | 0.113 | -0.282 | 0.184 | 0.397 |
| MDA | -0.719 | -0.134 | -0.103 | 0.504 | 0.172 | -0.129 | 0.060 | 0.387 |
| TPC | -0.694 | **0.218** | 0.119 | -0.256 | 0.244 | 0.556 | -0.099 | -0.116 |
| POD | -0.284 | **0.367** | 0.555 | 0.658 | -0.126 | 0.095 | -0.104 | -0.089 |
| APX | -0.553 | **0.222** | 0.258 | 0.017 | -0.585 | 0.151 | -0.439 | 0.145 |
| yield | -0.311 | **0.110** | -0.059 | 0.773 | 0.443 | 0.104 | 0.214 | -0.195 |
| **Heat** | **PC-I** | **PC-II** | **PC-III** | **PC-IV** | **PC-V** | **PC-VI** | **PC-VII** | **PC-VIII** |
| Eigenvalue | 9.845 | 5.646 | 5.035 | 3.875 | 3.130 | 2.766 | 1.576 | 1.127 |
| Variability (%) | 29.834 | 17.109 | 15.257 | 11.744 | 9.485 | 8.381 | 4.775 | 3.415 |
| Cumulative % | 29.834 | 46.943 | 62.200 | 73.944 | 83.428 | 91.810 | 96.585 | 100.000 |
| **Factor loadings:** | |  |  |  |  |  |  |  |
|  | **PC-I** | **PC-II** | **PC-III** | **PC-IV** | **PC-V** | **PC-VI** | **PC-VII** | **PC-VIII** |
| S.L | -0.032 | -0.644 | -0.347 | 0.619 | 0.182 | 0.208 | -0.032 | -0.055 |
| R.L | **0.703** | -0.274 | 0.214 | -0.504 | -0.300 | -0.108 | 0.075 | -0.155 |
| S.F.W | **0.521** | **0.725** | -0.110 | -0.173 | 0.337 | 0.106 | 0.006 | -0.189 |
| S.D.W | **0.559** | **0.168** | -0.558 | 0.496 | -0.154 | 0.277 | 0.022 | -0.030 |
| Sh.F wt | **0.339** | **0.817** | -0.270 | 0.020 | 0.338 | -0.031 | -0.007 | -0.168 |
| sh.d wt | **0.418** | **0.554** | -0.606 | 0.338 | 0.123 | -0.041 | -0.117 | -0.081 |
| R.F.W | **0.649** | **0.512** | 0.087 | -0.365 | 0.285 | 0.244 | 0.021 | -0.184 |
| R.D.W | **0.496** | -0.243 | -0.322 | 0.471 | -0.355 | 0.473 | 0.142 | 0.026 |
| T.Wp | **0.057** | -0.494 | -0.676 | -0.168 | -0.409 | 0.294 | 0.028 | 0.112 |
| O.p | -0.526 | -0.112 | 0.706 | 0.222 | -0.344 | 0.114 | 0.176 | -0.044 |
| Tr.P | -0.452 | **0.090** | 0.828 | 0.244 | -0.131 | -0.015 | 0.134 | -0.078 |
| R.W.C | **0.631** | **0.357** | 0.123 | -0.609 | 0.030 | 0.226 | -0.146 | 0.123 |
| FO | -0.610 | -0.005 | -0.722 | -0.206 | 0.047 | -0.061 | 0.234 | -0.055 |
| fm | -0.600 | -0.060 | -0.690 | -0.095 | -0.102 | -0.206 | 0.243 | -0.199 |
| Fv | -0.606 | -0.051 | -0.668 | -0.064 | -0.134 | -0.228 | 0.254 | -0.213 |
| tm | **0.074** | **0.472** | 0.473 | 0.536 | 0.245 | -0.032 | 0.263 | 0.361 |
| fv/fm | -0.208 | 0.206 | 0.209 | 0.493 | -0.651 | -0.399 | 0.010 | -0.213 |
| lyco | **0.887** | -0.238 | 0.063 | 0.187 | 0.036 | -0.329 | 0.069 | 0.064 |
| chl a | **0.807** | -0.469 | 0.078 | 0.027 | 0.228 | 0.169 | -0.177 | -0.093 |
| chl b | **0.798** | -0.207 | 0.052 | 0.215 | 0.037 | -0.504 | 0.121 | 0.050 |
| total chl | **0.861** | -0.296 | 0.063 | 0.179 | 0.093 | -0.353 | 0.047 | 0.014 |
| T.car | **0.935** | -0.277 | 0.019 | 0.133 | 0.098 | -0.146 | 0.002 | 0.016 |
| TOS | -0.331 | -0.460 | 0.274 | 0.101 | 0.677 | -0.225 | 0.265 | 0.116 |
| SOD | **0.184** | **0.466** | 0.112 | 0.627 | -0.552 | -0.009 | -0.120 | -0.156 |
| Pro | **0.163** | -0.488 | -0.373 | 0.342 | 0.325 | 0.412 | 0.314 | 0.325 |
| est | **0.224** | **0.465** | -0.289 | -0.357 | -0.447 | -0.203 | 0.121 | 0.517 |
| cat | **0.112** | -0.299 | 0.425 | -0.273 | -0.374 | 0.677 | 0.173 | -0.120 |
| protein | **0.041** | **0.653** | 0.148 | 0.324 | 0.197 | 0.105 | 0.622 | -0.089 |
| MDA | **0.656** | **0.098** | -0.231 | -0.076 | -0.291 | -0.604 | 0.226 | 0.029 |
| TPC | **0.470** | -0.147 | 0.157 | -0.416 | 0.049 | 0.188 | 0.657 | -0.301 |
| POD | -0.362 | -0.681 | 0.049 | -0.204 | 0.291 | -0.493 | -0.038 | -0.179 |
| APX | **0.702** | -0.289 | 0.114 | -0.346 | -0.470 | -0.107 | 0.202 | 0.136 |
| yield | -0.764 | **0.425** | -0.048 | -0.315 | -0.055 | -0.064 | 0.149 | 0.324 |
| **H+I** | **PC-I** | **PC-II** | **PC-III** | **PC-IV** | **PC-V** | **PC-VI** | **PC-VII** | **PC-VIII** |
| Eigenvalue | 8.143 | 7.224 | 4.986 | 3.821 | 3.055 | 2.154 | 1.644 | 0.972 |
| Variability (%) | 25.447 | 22.576 | 15.580 | 11.941 | 9.548 | 6.733 | 5.138 | 3.037 |
| Cumulative % | 25.447 | 48.023 | 63.603 | 75.544 | 85.093 | 91.825 | 96.963 | 100.000 |
| **Factor loadings:** | |  |  |  |  |  |  |  |
|  | **PC-I** | **PC-II** | **PC-III** | **PC-IV** | **PC-V** | **PC-VI** | **PC-VII** | **PC-VIII** |
| S.L | -0.296 | **0.532** | -0.272 | 0.384 | 0.605 | 0.014 | -0.176 | 0.102 |
| R.L | **0.055** | **0.091** | 0.062 | 0.726 | -0.545 | 0.254 | -0.307 | -0.039 |
| S.F.W | -0.297 | **0.844** | -0.087 | 0.028 | -0.015 | 0.422 | 0.096 | -0.059 |
| S.D.W | -0.340 | **0.466** | 0.736 | 0.286 | 0.172 | 0.027 | 0.121 | 0.001 |
| Sh.F wt | -0.474 | **0.780** | 0.028 | 0.122 | 0.181 | 0.251 | 0.234 | 0.019 |
| sh.d wt | -0.405 | **0.594** | 0.535 | 0.148 | 0.315 | 0.195 | 0.166 | 0.101 |
| R.F.W | -0.036 | **0.758** | -0.203 | -0.088 | -0.239 | 0.540 | -0.083 | -0.139 |
| R.D.W | -0.032 | -0.039 | 0.782 | 0.427 | -0.214 | -0.336 | -0.035 | -0.208 |
| T.Wp | **0.366** | -0.429 | 0.493 | -0.382 | 0.220 | 0.418 | -0.142 | 0.224 |
| O.p | -0.796 | -0.009 | -0.039 | -0.515 | 0.154 | 0.064 | -0.232 | -0.134 |
| Tr.P | -0.824 | 0.266 | -0.342 | -0.140 | -0.025 | -0.218 | -0.082 | -0.242 |
| R.W.C | -0.555 | **0.069** | 0.622 | -0.113 | -0.463 | 0.136 | -0.199 | -0.125 |
| FO | -0.094 | -0.643 | -0.562 | 0.216 | -0.147 | 0.015 | -0.026 | 0.439 |
| fm | -0.083 | -0.662 | 0.105 | -0.213 | 0.554 | 0.226 | 0.294 | -0.234 |
| Fv | **0.367** | -0.660 | 0.276 | -0.282 | 0.429 | 0.180 | 0.142 | -0.193 |
| tm | **0.287** | -0.011 | 0.811 | -0.293 | -0.117 | 0.337 | -0.069 | -0.206 |
| fv/fm | **0.792** | -0.473 | 0.285 | -0.210 | 0.035 | 0.029 | -0.141 | 0.052 |
| lyco | **0.872** | **0.445** | -0.014 | 0.034 | -0.004 | -0.151 | 0.110 | -0.076 |
| chl a | **0.626** | **0.550** | -0.236 | -0.125 | 0.174 | 0.113 | -0.433 | 0.063 |
| chl b | **0.775** | **0.225** | -0.016 | 0.143 | -0.190 | -0.291 | 0.413 | -0.191 |
| total chl | **0.860** | **0.465** | -0.148 | 0.018 | -0.020 | -0.119 | 0.010 | -0.085 |
| T.car | **0.814** | **0.527** | -0.040 | -0.022 | 0.215 | -0.015 | -0.103 | 0.027 |
| TOS | **0.587** | **0.416** | -0.302 | 0.319 | 0.130 | 0.442 | -0.209 | 0.182 |
| SOD | -0.065 | **0.448** | 0.374 | -0.529 | -0.580 | 0.123 | 0.155 | 0.017 |
| Pro | **0.739** | **0.434** | -0.272 | -0.090 | 0.024 | -0.298 | -0.143 | -0.272 |
| est | -0.847 | **0.007** | -0.367 | -0.076 | -0.291 | -0.152 | 0.128 | 0.136 |
| cat | -0.057 | **0.536** | 0.255 | -0.451 | 0.133 | -0.178 | 0.310 | 0.543 |
| protein | **0.331** | -0.434 | 0.147 | 0.566 | 0.145 | 0.371 | 0.446 | 0.034 |
| MDA | 0.000 | 0**.617** | -0.086 | -0.669 | 0.311 | -0.238 | 0.099 | 0.033 |
| TPC | -0.200 | -0.034 | -0.679 | 0.324 | 0.060 | 0.226 | 0.528 | -0.245 |
| POD | -0.067 | -0.551 | -0.621 | -0.325 | -0.039 | 0.338 | -0.273 | -0.104 |
| APX | -0.520 | **0.100** | 0.027 | 0.217 | 0.677 | -0.155 | -0.325 | -0.291 |
| yield | **0.224** | **0.117** | -0.619 | -0.534 | -0.301 | 0.246 | 0.282 | -0.194 |

S.L=Seedling Length: R.L= Root Length: S.F.W= Seedling Fresh Weight: Sh.FWt= Shoot Fresh Weight: Sh.DWt=Shoot Dry Weight: R.F.W=Root Fresh Weight: R.D.W=Root Dry Weight :T.Wp= Total Water Potential: O.P=Osmotic Potential, Tr.P= Turgor Potential, R.W.C= Relative Water Content: Fo *=*Minimal Fluorescence: Fm=Maximal  Fluorescence: Fv= Variable Fluorescence: Fv/Fm=Ratio: Lyco=Lycopene: Chla=Chlorophyll A: Chlb=Chlorophyll B: Total Chl=Total Chlorophyll: T.Car=Total Carotenoids: TOS=Total Oxidant Status: SD=Superoxide Dismutase: CAT=Catalase: TSP=Total Soluble Protein: MDA=Malondialdehyde: TPC=Total Phenolic Contents: POD=Peroxidase: APX=Ascorbateperoxidase
